# Overcoming Cytosolic Delivery Barriers of Proteins Using Denatured Protein-Conjugated Mesoporous Silica Nanoparticles

**DOI:** 10.1021/acsami.2c17544

**Published:** 2022-12-23

**Authors:** Julien Dembélé, Jou-Hsuan Liao, Tsang-Pai Liu, Yi-Ping Chen

**Affiliations:** † Graduate Institute of Biomedical Materials & Tissue Engineering, College of Biomedical Engineering, Taipei Medical University, Taipei 11031, Taiwan; ‡ Laboratory of Toxicology, Environment and Health, Doctorate School of Health, University Joseph Ki-Zerbo, Ouaga 03 BP 7021, Burkina Faso; § Department of Chemistry, National Taiwan University, Taipei 10617, Taiwan; ∥ Department of Surgery, Mackay Memorial Hospital, Taipei 10449, Taiwan; ⊥ Graduate Institute of Nanomedicine and Medical Engineering, Taipei Medical University, Taipei 11031, Taiwan; # International PhD Program in Biomedical Engineering, College of Biomedical Engineering, Taipei Medical University, Taipei 11031, Taiwan

**Keywords:** mesoporous silica nanoparticles, protein delivery, protein corona, TAT peptide, size/steric hindrance, surface functionalization

## Abstract

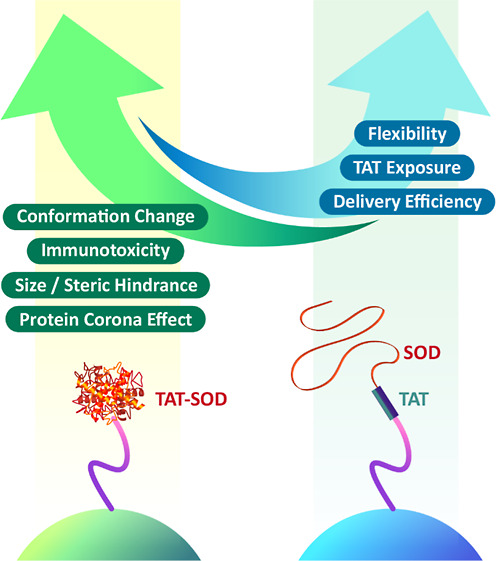

Intracellular delivery of therapeutic proteins has increased advantages over current small-molecule drugs and gene therapies, especially in therapeutic efficacies for a broad spectrum of diseases. Hence, developing the protein therapeutics approach provides a needed alternative. Here, we designed a mesoporous silica nanoparticle (MSN)-mediated protein delivery approach and demonstrated effective intracellular delivery of the denatured superoxide dismutase (SOD) protein, overcoming the delivery challenges and achieving higher enzymatic activity than native SOD-conjugated MSNs. The denatured SOD-conjugated MSN delivery strategy provides benefits of reduced size and steric hindrance, increased protein flexibility without distorting its secondary structure, exposure of the cell-penetrating peptide transactivator of transcription for enhanced efficient delivery, and a change in the corona protein composition, enabling cytosolic delivery. After delivery, SOD displayed a specific activity around threefold higher than in our previous reports. Furthermore, the in vivo biosafety and therapeutic potential for neuron therapy were evaluated, demonstrating the biocompatibility and the effective antioxidant effect in Neuro-2a cells that protected neurite outgrowth from paraquat-induced reactive oxygen species attack. This study offers an opportunity to realize the druggable possibility of cytosolic proteins using MSNs.

## 1 Introduction

The intracellular delivery of proteins into living cells presents an opportunity to become a new therapeutic approach to treat a broad range of human diseases, including genetic disorders, cancers, and so forth. Encompassing over 50% of a cell’s dry weight, proteins represent the most abundant macromolecules, playing critical effector roles in regulating almost all biological processes of cells.^1^ As to proteins’ advantages over small-molecule drugs, such as high specificity for targets and lower toxicity due to their natural molecular properties, protein delivery is considered a promising therapeutic strategy. Thus, there has been a recent proliferation of proteins in the drug development market.^2^ Additionally, direct intracellular delivery of functional proteins provides faster and more-efficient outcomes than gene therapy. It reduces off-target delivery beyond that of delivering DNA or RNA-encoded proteins, which rely on the transfection efficacy and the number of gene copies, such as in the ribonucleoprotein delivery for the clustered regularly interspaced short palindromic repeats-associated protein 9 (CRISPR/Cas9) gene editing.^3^ Such potential therapeutics offer alternatives against incurable diseases, such as genetic deficiencies of lysosomal enzymes known as lysosomal storage disorders that lead to lysosomal dysfunction.^4−6^

Current protein therapeutics in clinics mainly aim for extracellular targets, such as monoclonal antibodies, coagulation factors, and peptide hormones, whose receptors are expressed on cell membrane surfaces and secretory proteins. In contrast, intracellularly targeted proteins are barely “druggable”, which is attributed to the challenges of efficient intracellular delivery. Various strategies, including cell membrane disruption and the use of carriers, have been developed to overcome in vitro and in vivo delivery challenges.^7,8^ Recently, engineered nanoparticles (NPs) have been used to deliver therapeutic proteins, which is considered a promising approach toward effective protein therapy.^9^ Despite that progress, the efficacy of cytosolic protein delivery is still limited due to issues related to biological features and nanomaterials. Issues that restrict the delivery of therapeutic proteins into cells include the poor stability of the protein; low cellular membrane permeability; endosomal/lysosomal degradation; short half-live of the exogenous circulating proteins; and immunogenicity.^2,10,11^

Developing new techniques to overcome the limitations of protein delivery to the cytosol is needed.^7^ Over the past years, various kinds of NPs have been designed as carriers for therapeutic proteins to achieve intracellular delivery. As an attractive delivery approach, proteins are usually attached to the surface of NPs through direct covalent conjugation between the protein’s reactive moieties and functional groups on the NP’s surface. Most NP-mediated protein delivery systems employ a native protein for immobilization, so the protein purification process, the chemical solvent used for the conjugation procedure, and the stability of the protein’s environment may cause protein instability and conformational changes. The protein may have already lost its function before cellular delivery, and poor results or failure may be observed. Given the importance of the native conformation in retaining the protein’s active sites and biological activity, it is an indispensable prerequisite to design the immobilization of a protein on NPs. Therefore, keeping a protein’s conformation and stability after immobilization on NPs would greatly benefit protein delivery.^12,13^ In addition, evidence suggested that enzymatic NP immobilization with a chemical cross-linker was more efficient and preserved higher activity than direct immobilization, where the distance from the enzyme to the NP’s surface is shorter.^13,14^ The length of the chemical cross-linker is associated with the enzyme’s activity, indicating that longer cross-linkers may keep the enzyme far enough away from the NPs’ surface, allowing the enzyme to remain flexible without distorting its secondary conformation.

Furthermore, the phenomenon of the protein corona is considered an issue that can affect the efficiency of protein delivery and bioactivity.^15,16^ When exposed to biological fluids, NPs can rapidly adsorb proteins, forming a protein corona around the NPs. The protein corona layer can be conceptualized as “hard corona” and “soft corona”.^17^ Corona protein profiles vary depending on several characteristics of the NP, such as its size, charge, surface functionalization, and other properties.^18^ The protein corona establishes a new bio-interface between NPs and cells that may highly influence cellular internalization, cell viability, immune cell responses, and subsequent biological effects and biodistributions.^19,20^ Several plasma proteins composing the protein corona are ligands of receptors expressed on different cell surfaces and thus affect cellular internalization mechanisms.^21−24^ The protein corona can also annihilate the capacity of targeting moiety-functionalized NPs, leading to poor therapeutic efficacy.^25^ Understanding the protein corona composition of protein-conjugated NPs would provide insights into developing an approach for protein delivery and altering the protein’s fate.

In addition, it is also essential to understand the structural behavior of proteins conjugated or adsorbed onto NPs and their effects on protein delivery. It is well-known that the NP surface can cause a conformational change of conjugated proteins and passively adsorbed corona proteins and thus influence interactions with cells and subsequent biological events.^26,27^ According to previous reports, enzymes such as cytochrome *C*, lysozyme, and chymotrypsin might undergo secondary structural changes upon immobilization onto the NP surface with significant activity loss.^28^ Evidence suggested that the secondary structure of the corona proteins determined receptor-mediated cellular uptake.^29−31^ The secondary structure of a protein governs its biological activity. An irreversible change in a protein’s structure, induced by the chemical solvent used for the protein conjugation process or by the NP’s surface-altering conformational changes of the conjugated protein, may trigger protein aggregation and fiber formation, which could be harmful to cells. Thus, designing protein immobilization strategies to ensure a stable active conformation on the NP surface is very important.

Moreover, the abnormal unfolding of proteins on NP surfaces is responsible for the loss of function and the formation of novel conformational epitopes or exposure of “cryptic” epitopes that initiate immunological recognition and the production of neutralizing immunoglobulins.^32^ Hence, immunogenicity caused by the abnormal protein structures on NPs may impair the delivery efficacy, followed by decreased therapeutic efficacy. Delivery strategies are needed that prevent the abnormal unfolding of proteins.

Mesoporous silica NPs (MSNs) are promising nanomaterials that have attracted much attention in biochemical and pharmaceutical applications such as therapeutics delivery, bioimaging, and biosensing. Owing to their easy functionalization and mesoporous features, MSNs can be tuned to accommodate many types of therapeutics, including small molecules, proteins/enzymes, antibodies, nucleic acids, and tracking agents, that have significant advantages in cancer therapy and diagnosis, enzyme replacement therapy, gene editing, tissue engineering, and so forth.^33−37^ In previous studies,^38,39^ we demonstrated the effectiveness of a denatured enzyme delivery system, employing MSNs and cell-penetrating peptide transactivator of transcription (TAT) fusion proteins produced by a genetic engineering approach. In vitro experiments revealed that the single delivery of the antioxidant enzyme, superoxide dismutase (SOD), or co-delivery of two enzymes, SOD and glutathione peroxidase, with a cascade reaction, could be successfully carried out. The protein/enzyme in a denatured form can be refolded inside cells, and its enzymatic activity restored, allowing protection from attack by reactive oxygen species (ROS). The strategy takes advantage of (1) TAT peptides with positively charged amino acids, which can provide enhanced delivery and avoid the entrapment by endosomes/lysosomes via non-endocytosis uptake, and (2) a protein’s denatured state, characterized by high Gibbs free energy and reduced conformational hindrance, allowing it to be translocated independently of cellular energy consumption.^40,41^ The delivery strategy opens a new window for developing potent cytosolic protein therapeutics. These exciting results simultaneously include easy therapeutic protein production, smart conjugation, purification, efficient cytosolic delivery of the protein by non-endocytosis without endosomal/lysosomal trapping, and protein refolding with increased activity.

In fact, this delivery method is useful but needs further validation using in vitro and in vivo models. Many detailed studies of the parameters affecting protein delivery efficacy are uncompleted; however, they are highly relevant to develop a protein delivery approach. In the present study, we report an improved method for cytosolic delivery of a denatured protein using MSNs toward in vivo use. The fluorescent dye rhodamine isothiocyanate isomer (RITC) incorporated MSNs (RMSNs), functionalized with polyethyleneimine (PEI) molecule, polyethylene glycol (PEG) cross-linker, and Ni-nitrilotriacetic acid (NTA)-chelated ligand, were conjugated with the TAT peptide fusion SOD protein via metal coordination. The purpose of the design strategy includes: (1) increasing the protein flexibility and decreasing irreversible changes in the protein structure caused by the NP surface, through more-extended cross-linker modification, (2) introducing PEG to enhance the biocompatibility and stability, as well as to reduce the protein corona effect, clearance from the bloodstream by the mononuclear phagocyte system and off-target accumulation in organs after administration,^42−45^ and (3) using positively charged PEI modification which contributed to cell uptake and the proton sponge effect for endosomal escape.^46^ PEG with short chain can enhance the antifouling effect, achieving the purpose of preventing aggregation of NPs during the bare MSN synthesis. The cross-linker with a longer PEG chain (MW 3.4k) linked the MSNs and SOD and created a distance that benefited SOD’s flexibility and activity. Importantly, we specifically demonstrated that the new method could increase enzymatic activity by around threefold compared to our previous results. Then, fundamental exploration of the RMSN-Ni-TAT-denatured SOD (SODd) and RMSN-Ni-TAT-native SOD (SODn) was performed, focusing on studies of the protein corona effect regarding their cellular internalization and SOD activity, the molecular mechanism of cell uptake, the role of the TAT peptides, and a protein secondary structure analysis. Furthermore, protein corona formation was visualized by transmission electron microscopy (TEM) imaging and identified by liquid chromatography–tandem mass spectroscopy (LC–MS/MS). We clarified that the new denatured protein delivery takes advantage of reduced size/steric hindrance, an absence of protein conformational change issues, the ability to avoid protein corona adsorption, and a clear exposure of the TAT peptide, thus leading to effective delivery. The biosafety evaluation of RMSN-Ni-TAT-SOD, including its biodistribution, histopathological analysis, and hematotoxicity, was verified in BALB/c mice. To our knowledge, this is the first study to perform an in vivo toxicity evaluation and a comparative exploration of detailed mechanisms on the same protein in native and denatured states when conjugated onto NPs. Instead of the limited efficiency gene therapy and low therapeutic efficacy of small-molecule drugs for neuron therapy, MSN-mediated denatured protein delivery can be an attractive therapeutic approach to protect neuron cell outgrowth against ROS-induced damage. This study offers an opportunity to realize the druggable possibility of cytosolic proteins using MSNs, allowing us to tackle more-difficult diseases in the future.

## 2 Materials and Methods

### 2.1 Chemicals and Reagents

Chemicals used in the present study were obtained from commercial suppliers and used without further purification. Ammonium bicarbonate (for analysis, 99%), ammonium hydroxide (ACS reagent, 28–30% solution in water), cetyltrimethylammonium bromide (CTAB, 99%+), tetraethyl orthosilicate (TEOS, 98%), 3-aminopropyltrimethoxysilane (APTMS, 95%), and zinc chloride (99.99%, cell culture tested) were purchased from Acros. Aldehyde dehydrogenase (ALDH4) potassium-activated from baker’s yeast, bovine serum albumin (≥98%), copper(II) chloride hydrate (cell culture tested), iodoacetamide (IAA), RITC (70%), *N*_α_,*N*_α_-bis(carboxymethyl)-l-lysine (BCLH, 97%), trypsin from bovine pancreas (salt-free lyophilized powder), urea (98+%, molecular biology tested), and 2-iminothiolane chloride (Traut’s reagent, 98%) were purchased from Sigma-Aldrich. Acetonitrile (ACN) and trifluoroacetic acid (TFA) were purchased from Fisher Scientific. Nickel(II) chloride hexahydrate (98%) was purchased from Alfa Aesar. 1,4-Dithiothreitol (DTT) was purchased from Roche. Maleimide (MAL)-PEG-succinimidyl ester (SCM) (MAL-PEG-SCM, MW 3.4k) was purchased from the Creative PEGWorks Biotechnology Company. ZipTip C18 was from Millipore. Ampicillin, imidazole (biotechnology grade), LB broth Lennox, isopropylthio-β-galactoside (IPTG) (BioUltraPure grade), and sodium phosphate dibasic (anhydrous, ACS grade) were purchased from BioShop. 2[Methoxy(polyethyleneoxy)propyl]trimethoxysilane (PEG-silane, 90%) and trimethoxysilylpropyl modified (PEI) (PEI-silane, 50%, MW 1500–1800) were purchased from Fluorochem. The WST-1 cell proliferation reagent was purchased from Clontech.

### 2.2 Synthesis of RMSN-PEG/PEI

The synthesis of 50 nm RITC-conjugated MSNs (RMSN) was based on previous reports.^38,47^ Briefly, 150 mL of 0.128 M of an aqueous ammonia solution was prepared to dissolve 0.29 g of CTAB as a surfactant and was stirred continuously for 15 min in a water bath at 50 °C. APTMS-conjugated RITC (2.5 mL) was added, followed immediately by the dropwise addition of 2.5 mL of 0.88 M TEOS diluted in 99.5% ethanol under continuous stirring for 1 h. After that, 20 μL of PEI-silane and 550 μL of PEG-silane diluted in ethanol were introduced for surface modification and stirred for 30 min. The NPs were aged for 24 h in a water bath at 50 °C and subjected to hydrothermal treatment in an oven for 24 h at 70 and 90 °C, respectively. The surfactants were removed by hydrochloric acid extraction at 60 °C, followed by washing several times with ethanol on a cross-flow filtration system. Finally, RMSN-PEG/PEI was obtained and stored in 99.5% ethanol.

### 2.3 Synthesis of RMSN-Ni

For 6× His-tag protein conjugation, nickel-NTA (Ni-NTA) was introduced onto the RMSN-PEG/PEI surface. The heterobifunctional crosslinker of MAL-PEG-SCM (MW 3.4k) was covalently conjugated with RMSN-PEG/PEI through the SCM group by reacting with the amine group of PEI from RMSN-PEG/PEI. First, 13.6 mg of the MAL-PEG-SCM crosslinker was dissolved in 5 mL of 1× phosphate-buffered saline (PBS) and gently added to 40 mg of RMSN-PEG/PEI dispersed in 5 mL of 1× PBS, under continuous stirring for 2 h at room temperature to form RMSN-PEG-MAL. Then, RMSN-PEG-MAL was centrifuge-washed with 1× PBS to remove the unreacted MAL-PEG-SCM and was then dispersed in 10 mL of 1× PBS. Meanwhile, 10.49 mg of BCLH, and 13.7 mg of Traut’s reagent (TLC) were, respectively, dissolved in 10 and 1 mL of 1× PBS and reacted for 30 min at room temperature to obtain the thiolated complex of BCLH–TLC. Then, 10 mL of the BCLH–TLC complex was added to 10 mL of RMSN-PEG-MAL for reaction overnight at 4 °C. The BCLH–TLC complex was covalently conjugated with RMSN-PEG-MAL through its thiol group to react with the MAL group of RMSN-PEG-MAL. After centrifugation and washing with double-distilled water (ddH_2_O) several times to remove unreacted chemicals, the intermediate product called RMSN-PEG-TLC–BCLH was collected. After that, RMSN-PEG-TLC–BCLH dispersed in 10 mL of ddH_2_O was mixed with 200 μL of 500 mM NiCl_2_ and stirred for 4 h at room temperature. The coupling is based on nickel coordinating with the three carboxyl groups of BCLH. The final product, called RMSN-Ni, was collected by centrifugation and washed with ethanol several times to remove the unreacted NiCl_2_. Finally, RMSN-Ni was dispersed in 99.5% ethanol and stored at room temperature.

### 2.4 Expression of Recombinant TAT-SOD Proteins

Construction of the pQE-TAT-SOD plasmid and the JM109 strain for fusion protein production were based on our previous report.^38^ In general, the TAT-SOD protein was overexpressed by IPTG induction and then extracted by ultrasonication on ice. The collected lysates containing 6× His-TAT-SOD protein were confirmed by 12% sodium dodecylsulfate polyacrylamide gel electrophoresis (SDS-PAGE) for subsequent conjugation.

### 2.5 Synthesis of Native and Denatured Forms of TAT-SOD-Conjugated MSNs (RMSN-Ni-TAT-SOD)

For immobilization of the native form of TAT-SOD, RMSN-Ni was directly added to crude lysates that contained 6× His-TAT-SOD at 4 °C for 2 h. After that, TAT-SOD in its native form conjugated to MSNs (also called RMSN-Ni-TAT-SODn) was isolated and washed by centrifugation, resuspended in ddH_2_O three times, and stored in ddH_2_O at 4 °C. To ensure the same amount of protein immobilization, half of the RMSN-Ni-TAT-SODn was dissolved in 8 M urea with continuous stirring for 2 h at room temperature to obtain TAT-SOD in a denatured form conjugated to MSNs (also called RMSN-Ni-TAT-SODd). After washing with ethanol and centrifugation, RMSN-Ni-TAT-SODd was collected and stored in 99.5% ethanol at 4 °C to maintain the denaturation.

### 2.6 Characterization of MSNs

TEM imaging was used to observe the morphology and mesoporous channels of MSNs at 75 kV (Hitachi H-7100). Pore sizes of MSNs were measured using an N_2_ adsorption–desorption isotherm based on Brunauer–Emmett–Teller and Barrett–Joyner–Halenda calculation methods. The Zetasizer Nano ZS (Malvern, UK) was used to determine the hydrodynamic size by dynamic light scattering (DLS) and the ζ potential by electrophoretic mobility in different solutions. The amount of nickel in the sample was analyzed by an inductively coupled plasma mass spectrometer (Agilent 7800 ICP–MS).

### 2.7 Circular Dichroism Measurements

The secondary structure of TAT-SOD was measured using a circular dichroism (CD) analysis according to a previous report.^48^ Basically, 2.07 μM of protein was dispersed in sodium phosphate buffer (50 mM), and CD spectra were recorded on a Jasco J-715 spectropolarimeter. Data were analyzed using CDSSTR software to estimate the protein’s secondary structure. The mean residue ellipticity was calculated based on the mean residual weight estimated from the protein’s primary structure.

### 2.8 Cell Culture

HeLa cells, a human epithelial cervical cancer cell line, and the Neuro-2a (N2a) mouse neuroblast cell line derived from neuroblastomas, obtained from the American Type Culture Collection (ATCC, Manassas, VA), were cultured in Dulbecco’s modified Eagle medium (DMEM) (Gibco) supplemented with 10% fetal bovine serum (FBS; Gibco) and 100 U/mL of penicillin and streptomycin (Gibco) at 37 °C under 5% CO_2_ atmospheric conditions. At 80% confluence, 0.25% trypsin was used to detach the cells for passaging.

### 2.9 Cellular Uptake of MSNs Using Flow Cytometry

The cellular uptake efficiency of NPs by HeLa cells was analyzed by FACSCalibur flow cytometry (BD Biosciences) to quantitatively detect the red-emitting fluorescein dye of RITC conjugated onto the MSNs. First, HeLa cells were seeded in six-well plates at a density of 2 × 10^5^ cells/well for 24 h. Next, various concentrations of RMSN-Ni-TAT-SODn and RMSN-Ni-TAT-SODd (25–500 μg/mL) were used to treat cells in serum-containing and serum-free media conditions for 4 h; then, the cells were washed twice with 1× PBS to remove non-uptaken NPs and harvested by trypsinization. Finally, cells were collected by centrifugation and analyzed using flow cytometry.

### 2.10 Western Blot Analysis

Total cell lysates (50 μg) were prepared to separate proteins on 10% SDS-PAGE. Proteins were transferred electrophoretically to a polyvinylidene difluoride membrane, which was blocked by 5% (w/v) non-fat milk in a blocking buffer (1× Tris-buffered saline with 0.1% Tween 20 detergent) for 1 h. Then, the polyvinylidene fluoride membrane was incubated with primary antibodies against α-tubulin (Oncogene Science; 1:300,000), SOD (GenScript; 1:36,000), p-p38 (Santa Cruz; 1:500), and GAPDH (Abcam; 1:10,000) at 4 °C overnight. Afterward, the polyvinylidene difluoride membrane was washed and then incubated with a horseradish peroxidase-conjugated immunoglobulin G secondary antibody (Santa Cruz, 1:2000 dilution) for 1.5 h at room temperature. According to the manufacturer’s instructions, an enhanced chemiluminescence substrate kit (Amersham Pharmacia Biotech) was used to visualize the bands.

### 2.11 Determination of SOD Activity

A SOD assay kit (Cayman Chemical) was used to determine the specific enzymatic activity of SOD based on the principle of the detection of a tetrazolium salt formed during the generation of superoxide radicals by xanthine oxidase and hypoxanthine. The measurement followed a modified protocol of McCord and Fridovich.^49^ The enzymatic activity of SOD was expressed as units per milligram (U/mg) of total proteins (TPs), where a unit of enzyme is the amount required for the dismutation of 50% of the superoxide radical.

### 2.12 Endocytosis Pathway Analysis

HeLa cells were pretreated for 1 h with various chemical inhibitors of endocytosis pathways, including sodium azide (10 mM), 2-deoxyglucose (6 mM), filipin III (5 μg/mL), chlorpromazine (CPZ) (30 μM), and amiloride (10 μM). After blocking different endocytosis routes, cells were exposed to RMSN-Ni-TAT-SODn and RMSN-Ni-TAT-SODd at a concentration of 500 μg/mL in serum-containing and serum-free media conditions with endocytosis inhibitors for another 4 h. After that, cells were washed with PBS and trypsinized for a FACSCalibur flow cytometer (BD Biosciences) analysis.

### 2.13 Formation of an In Vitro Protein Corona

To assess the in vitro protein corona formation, 0.5 mg of MSN-Ni-TAT-SODn or MSN-Ni-TAT-SODd dispersed in PBS was mixed with DMEM supplemented with 10% FBS to a final volume of 1 mL. To mimic dynamic in vivo conditions, the mixture was incubated on an orbital shaker at 250 rpm for 30 min at room temperature to allow the proteins to be absorbed onto the surface of the NPs. Then, through centrifugation at 15,570*g* for 30 min and washing with 1× PBS three times at 25 °C to remove the unbound and loosely bound proteins, the complex NP-protein corona was isolated for the following experiments.

### 2.14 Quantification of the Protein Corona

Amounts of the protein corona adsorbed onto both the MSN-Ni-TAT-SODn and MSN-Ni-TAT-SODd formulations were quantified using a bicinchoninic acid (BCA) protein kit following the manufacturer’s instructions. The sample was mixed with the BCA reagent and then incubated at 60 °C on a heat block for 30 min. The absorbance was measured at 562 nm on a UV–vis spectrophotometer (Thermo Fisher BioMate 3S). The protein concentration was estimated by comparing to a standard curve, and data are presented in triplicate.

### 2.15 TEM Visualization of the Protein Corona Layer

The complex of MSN-Ni-TAT-SOD and protein corona was dispersed in ddH_2_O to a concentration of 0.05 mg/mL. Then, 10 μL of the sample was placed onto a carbon-coated copper grid for 60 s, and excess liquid was removed using absorbent paper. Negative staining was performed by applying a drop of 10 μL of uranyl acetate (2%) for 60 s, and absorbent paper was used to remove the excess uranyl acetate. After drying for 10 min, images of the protein corona were taken with a Hitachi TEM HT 7700.

### 2.16 Protein Corona Composition Identification by LC–MS/MS

The complex of MSN-Ni-TAT-SOD and the protein corona was mixed with 6× SDS-PAGE loading buffer containing 2% (w/v) SDS and DTT (50 mM), denatured by heating at 100 °C for 10 min, and then loaded on a 12% SDS-PAGE gel. After electrophoresis, the gel was stained with Coomassie brilliant blue R-250 dye and washed with ddH_2_O overnight. Bands of corona proteins were imaged. For in-gel digestion, the proteins bands were excised from the gel, cut into small 1.5 mm pieces, and then subjected to washing with a gradated ratio (1:1; 1:0.85; 1:0.8) of ammonium bicarbonate buffer (ABC buffer, 25 mM) and ACN (100%) until the blue dye had completely been destained. To reduce disulfide bonds, the gel was first treated with a DTT solution (10 mM) for 1 h in a water bath at 60 °C. After washing with 100% ACN to remove the unreacted DTT, the gel was treated with a solution of IAA (55 mM) for 45 min at room temperature while protected from light. The unreacted chemicals were removed by washing with ACN, followed by drying in a vacuum concentrator. Then, 0.8 ng/μL of a trypsin solution was added to cover the gel pieces, which were incubated at 37 °C for 16 h. After digestion, the proteins were extracted five times with 100 μL of TFA (0.1%) in ABC/ACN buffer (1:1) and dried in a vacuum concentrator for 1 day. After the sample had dried, it was stored at −20 °C until the subsequent desalting process.

For the protein corona analysis by LC–MS/MS, the sample was dissolved in 20 μL of a TFA (0.1%) solution and desalted using C18 ZipTips. Finally, the sample was eluted with 20 μL of 50% (v/v) TFA (0.1%) and ACN and dried in a vacuum concentrator for 1 h. Corona proteins were analyzed by label-free quantification on an Orbitrap Fusion Lumos Tribrid Mass Spectrophotometer (Thermo Fisher Scientific). Data were searched against the mouse protein database. Confident protein identification was filtered based on the following criteria: (1) the protein false discovery rate was set to “high”; (2) “IsMasterProtein” was chosen; and (3) the Mascot score was set to ≥25. Finally, the relative abundance of each protein was calculated according to the following eq 1
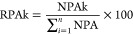
1where RPAk is the relative abundance of protein k, NPA is the normalized protein abundance, and NPAk is the normalized abundance of protein k.

### 2.17 Ingenuity Pathways Analysis

The experimental fold changes of relative protein abundances between the protein coronas of RMSN-Ni-TAT-SODd and RMSN-Ni-TAT-SODn were determined according to the following eq 2
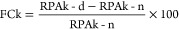
2where FCk is the fold change of protein k, RPAk-d is the relative abundance of corona protein k of RMSN-Ni-TAT-SODd, and RPAk-n is the relative abundance of corona protein k of RMSN-Ni-TAT-SODn. QIAGEN Ingenuity Pathway Analysis Online Software was used to analyze the fold changes of corona proteins. A core analysis was run, and enriched canonical pathways by fold changes of corona proteins were generated. Pathway enrichment was rated following *p* values, and the determined *Z*-scores were classified by different colors (orange, white, blue, and gray) to indicate pathway activation. The significantly upregulated and downregulated corona proteins were also determined.

### 2.18 In Vivo Biodistribution

Balb/c mice (8 weeks old, *n* = 3) were euthanized at 24 h post-intravenous (IV) injection with RMSN-Ni and RMSN-Ni-TAT-SOD (50 mg/kg). Images of harvested main organs, including the heart, liver, spleen, lungs, and kidneys, were acquired with an in vivo imaging system (IVIS) (Xenogen IVIS-200) to evaluate the biodistribution of various MSNs by detecting the fluorescence signals of RMSNs.

### 2.19 Histological Analysis

At the endpoint of the same treatment, harvested organs were fixed in 10% formalin and then sliced and stained with hematoxylin and eosin (H&E) to assess the toxicity. After that, histological imaging analysis of cryostat sections of frozen tissues was conducted using microscopy (Olympus IX-71).

### 2.20 Biosafety Assessment

Mice were injected intraperitoneally with RMSN-Ni and RMSN-Ni-TAT-SOD at a concentration of 50 mg/kg. At 24 h post-injection, the blood of mice was collected by cardiac puncture, and complete blood count (CBC) and serum biochemical analyses were assayed at the Taiwan Mouse Clinic (National Comprehensive Mouse Phenotyping and Drug Testing Center).

### 2.21 Neuron Differentiation and Protection

N2a cells were cultured in six-well plates at a density of 3 × 10^5^ cells/well for 24 h. Following treatment with RMSN-Ni-TAT-SODn (equivalent to 5 and 25 μg of SOD) for 4 h, cells were washed twice with 1× PBS to remove any non-uptaken NPs and were then treated with or without 20 μM of retinoic acid (RA) and 30 μM *N*,*N*′-dimethyl-4,4′-bipyridinium dichloride (paraquat, PQ). Afterward, neurite outgrowth and neurite formation were imaged on days 2 and 5 using microscopy. With the same treatment on day 8, cells were fixed in 4% paraformaldehyde and washed with 1× PBS. Then, the cytoskeleton and nuclei were, respectively, stained with Alexa Fluor 488-labeled phalloidin (green color) and 4′,6-diamidino-2-phenylindole (DAPI) (blue color). The red color indicated the RITC signal from RMSN-Ni-TAT-SODn. Images of neuron differentiation and protection were captured using fluorescence microscopy.

### 2.22 Superoxide Detection

Following the same treatment, N2a cells were washed with 1× PBS, and then 5 μM of dihydroethidium (DHE) was used to stain intracellular ROS, which were detected and quantified using flow cytometry.

### 2.23 Statistical Analysis

Values are given as the mean ± standard deviation (SD). Statistical analyses were performed by Student’s *t*-test. *p < 0.05 was considered a statistically significant difference, whereas **p < 0.01 and ***p < 0.001 indicated very significant and highly significant differences, respectively.

## 3 Results and Discussion

### 3.1 Synthesis and Characterization of MSNs

**Figure 1:**
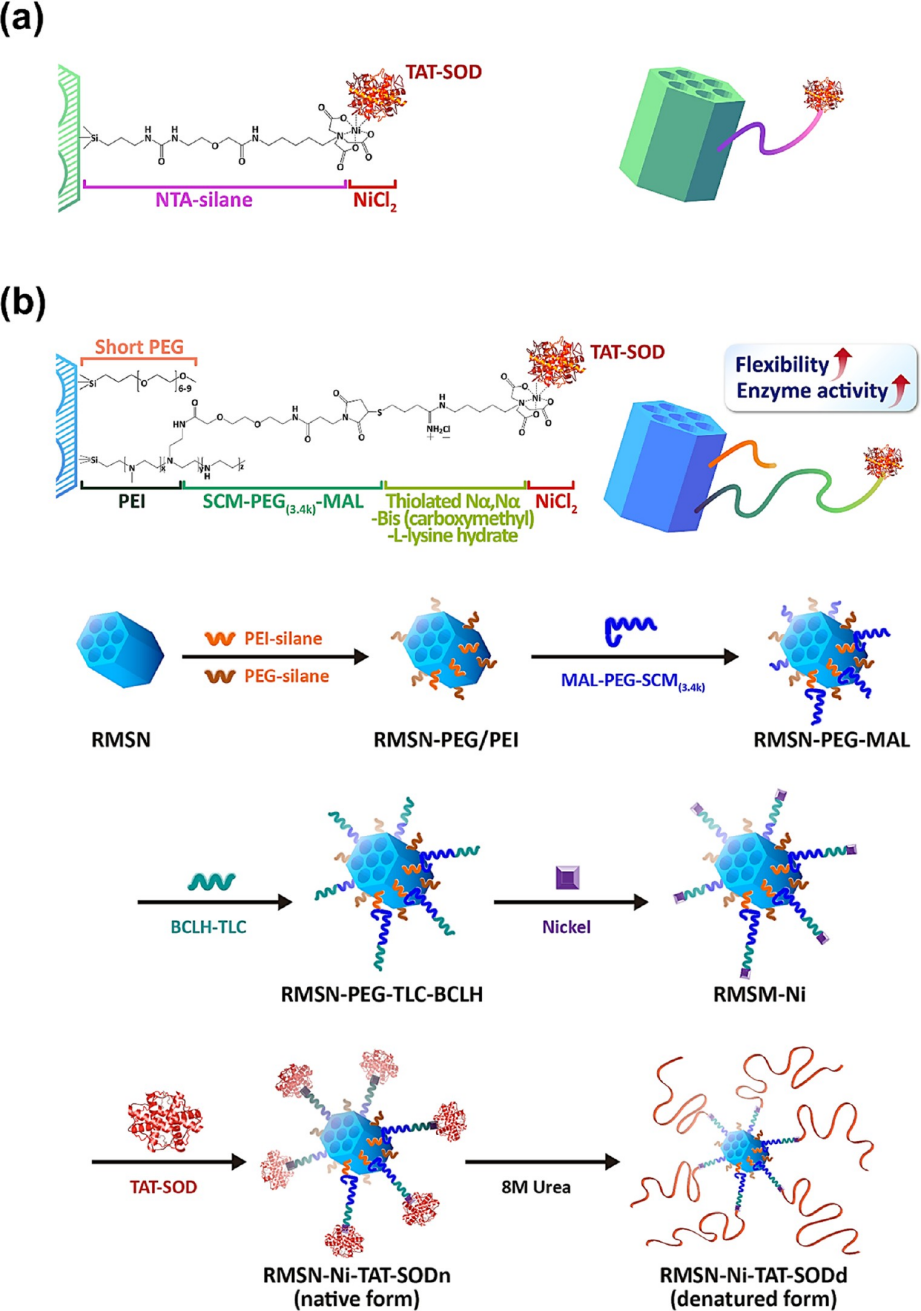
Synthesis of RMSN-Ni-TAT-SOD. (a) Schematic illustration of our previous design for the chemical conjugation of TAT-SOD with MSN-Ni. MSN-Ni was produced by co-condensation of MSN with NTA-silane, followed by the nickel coupling. Next, TA-SOD with 6× His-tag was conjugated to MSN-Ni via metal affinity binding. (b) Schematic illustration of the current design and synthesis procedure of RMSN-Ni-TAT-SOD. The detailed conjugation process was described in the method.

The synthesis process of RMSN-Ni-TAT-SOD is illustrated in Figure 1. The design is based on the interaction between the 6× His-tag protein and nickel through metal chelate coordination. The current design shown in Figure 1b was modified according to our previous reports (Figure 1a),^38,39^ aiming for in vivo use. (1) RMSN-PEG/PEI was synthesized by co-condensation of RMSN with PEG-silane and PEI-silane. (2) The heterobifunctional crosslinker of MAL-PEG-SCM with MW of 3.4k was covalently conjugated with RMEN-PEG/PEI to form RMSN-PEG-MAL. (3) BCLH–TLC was grafted onto the MAL group of RMSN-PEG-MAL to obtain RMSN-PEG-TLC-BCLH. (4) RMSM-Ni was produced by coupling nickel through its coordination with three carboxyl groups of RMSN-PEG-TLC-BCLH. (5) TA-SOD with 6× His-tag was conjugated to RMSN-Ni via metal affinity binding to obtain RMSN-Ni-TAT-SODn (n indicates that TAT-SOD was in its native form). (6) RMSN-Ni-TAT-SODd (d indicates that TAT-SOD was in its denatured form) was prepared using 8 M urea.

**Figure 2:**
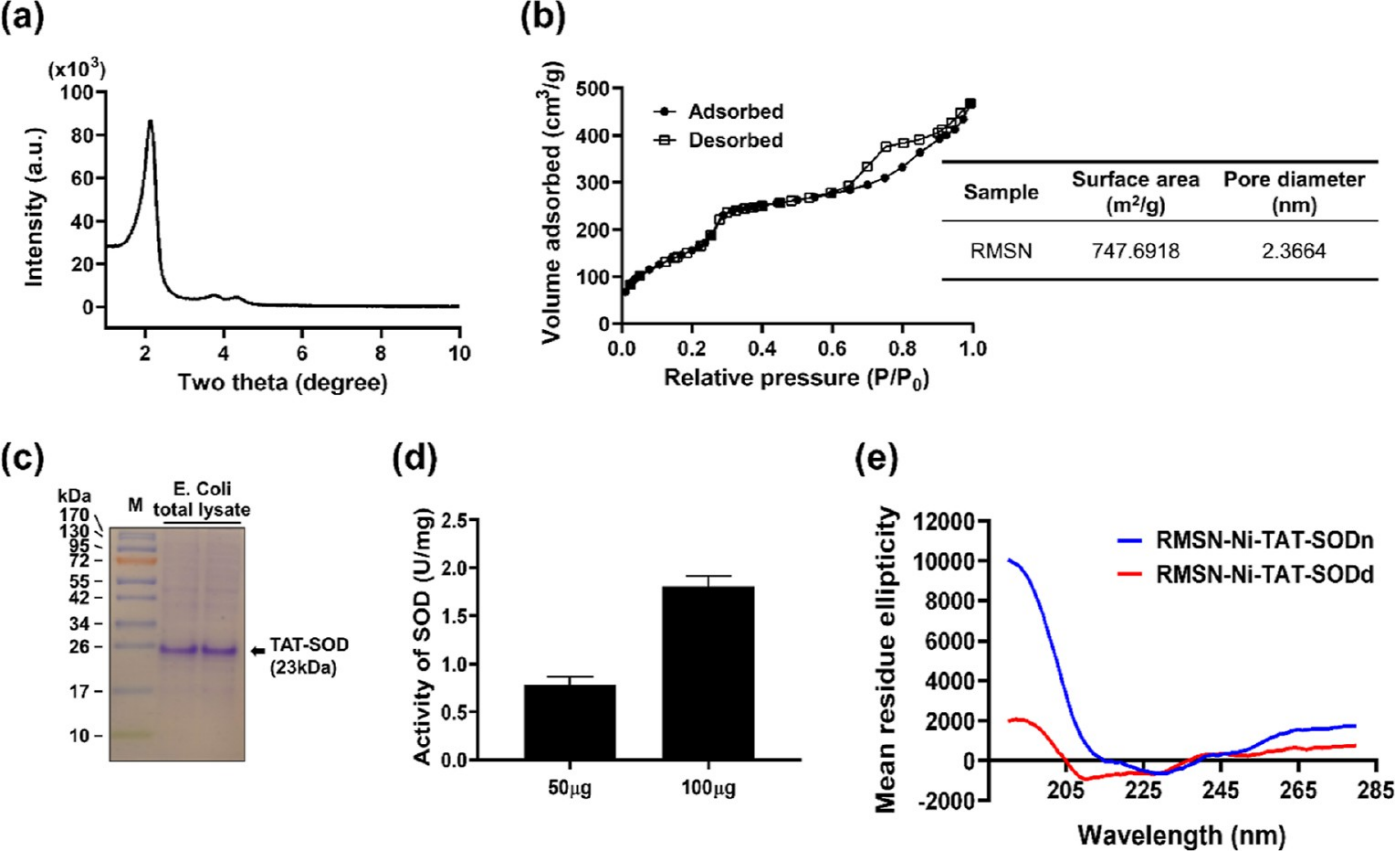
Characterization of the porous structure of RMSN and human recombinant SOD fused with TAT peptide produced by an expression system. After the synthesis of RMSN, the pore structure and the pore size were analyzed by (a) X-ray diffraction (XRD) patterns and (b) Brunauer–Emmett–Teller, respectively. (c) The recombinant TAT-Cu, Zn SOD was overexpressed in by IPTG induction and assayed by electrophoresis of 12% SDS-PAGE. (d) Enzymatic activity of SOD in crude lysates. (e) CD spectroscopic analysis of SOD’s secondary structure. Native and denatured forms of TAT-SOD were dissociated from the surface of MSNs using 500 mM imidazole for the CD spectroscopic analysis. Blue: SODn dissociated from RMSN-Ni-TAT-SODn; red: SODd dissociated from RMSN-Ni-TAT-SODd.

RMSN-Ni-TAT-SOD was synthesized as described in detail in the experimental section. The RMSNs with a 50 nm diameter were first synthesized and characterized. To investigate the structural order of RMSN, the pore structure was analyzed by XRD. As shown in Figure 2a, the pattern revealed a typical 2D hexagonally ordered structure of RMSN. Afterward, an N_2_ adsorption–desorption analysis was employed to determine the size of the pore diameters and the surface area. Results exhibited type VI isotherms, concordant with previous studies,^47,50^ with highly uniform cylindrical pores of the MSNs. The pore size and surface area of RMSN were around 2.4 nm and 750 m^2^/g, respectively (Figure 2b).

Following our previous method,^51^ the JM109 was transfected with an expression vector encoding 6× His-tag-human recombinant Cu, Zn-SOD (SOD1) fused with the TAT peptide. With IPTG induction of the expression of the protein of interest for 3 h, cells were harvested and lysed. Overexpression of the TAT-SOD protein was confirmed from lysates by a 12% SDS-PAGE analysis (Figure 2c), indicating that the majority of the expressed TAT-SOD had a MW of around 23 kDa. The crude lysate showed dose-dependent enzymatic activity of SOD (Figure 2d). Native TAT-SOD from lysates was then directly conjugated onto RMSN-Ni through the coordination of 6× His-tag and nickel to form RMSN-Ni-TAT-SODn for the following experiments. Under the same conditions, RMSN-Ni-TAT-SODn was further processed for protein denaturation to ensure the same amount of TAT-SOD immobilization. Under treatment with 8 M urea overnight at room temperature, the denatured form of TAT-SOD-conjugated MSNs, called RMSN-Ni-TAT-SODd, was obtained, followed by washing with ddH_2_O to remove the urea. As shown in Figure 2e, CD spectroscopic measurements confirmed the denaturation of TAT-SOD, revealing a change in its secondary structure, indicated by a significant decrease in the ratio of the beta-sheet and an increase in the beta-turn compared to the native structure of TAT-SOD.

**Figure 3:**
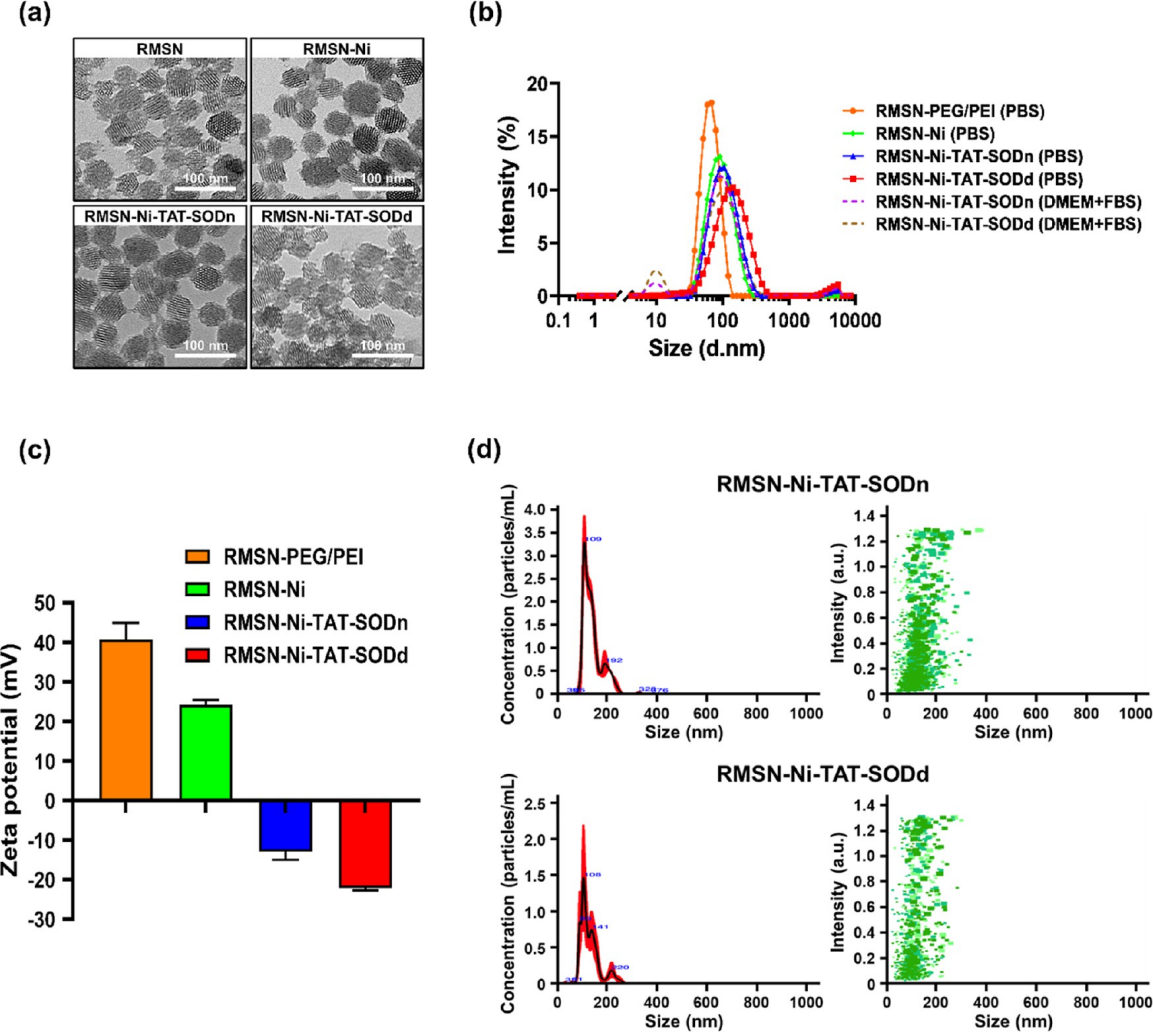
Characteristics of various RMSN derivatives. (a) TEM images. The NPs were deposited on carbon-coated copper grids and dried at room temperature for TEM observation. Scale bar: 100 nm. (b) Hydrodynamic size distribution. RMSN derivatives were dispersed in PBS (0.2 mg/mL) and culture medium supplemented with 10% FBS (0.5 mg/mL) and their particle size was measured by DLS. (c) ζ potential analysis. 0.5 mg/mL of various RMSN derivatives were dispersed in ddH_2_O for the measurement of surface charge. (d) NTA of the hydrodynamic size distribution and the concentration of RMSN-Ni-TAT-SODn and RMSN-Ni-TAT-SODd. The NPs were dispersed in PBS (1 × 10^–12^ mg/mL).

TEM, DLS, and NTA were used to characterize MSNs and their derivatives. The morphology and mesopore structure of MSN derivatives were observed in TEM images, suggesting that all MSNs exhibited well-ordered hexagonal structures with uniform mesoporous channels (Figure 3a). Comparing images of MSNs and their derivatives, no significant morphological differences indicated that the processes of chemical modification and protein conjugation had not destroyed their architecture. DLS measurements demonstrated that all the MSN derivatives had a well-dispersed size distribution with an average hydrodynamic diameter ranging from 61 to 122 nm in PBS and DMEM + 10% FBS (Figure 3b and Table S1). Owing to protein conjugation, the sizes of RMSN-Ni-TAT-SODn and RMSN-Ni-TAT-SODd showed an increase compared to MSN-PEG/PEI; however, they were smaller than those in our previous report,^38,39^ which may have been due to incorporation of PEG for surface passivation.^35,44^ The particle’s size influences cell uptake, and the present data meet the requirements for biological exploration.

The ζ potential measurement of MSN-PEG/PEI presented a positive charge of +40.7 mV owing to the modification by PEI with its amine groups (Figure 3c, Table S1). The size and ζ potential of MSN-PEG/PEI were further monitored for 3 weeks after synthesis, demonstrating their good stability and reproducibility (Table S2). Afterward, RMSN-PEG/PEI was modified with nickel (RMSN-Ni) to allow the conjugation of the 6× His-tag antioxidant enzyme SOD. The success of surface modification was monitored by measuring the ζ potential after applying different chemical linkers and nickel conjugations. As shown in Figure S1, MAL-PEG-SCM crosslinked to form RMSN-PEG-MAL (RMSN-PEG/PEI + MAL-PEG-SCM), which decreased the ζ potential from +40.7 to +10.4 mV, which may have been due to the reduced number of free terminal amine groups of PEI, which reacted with the SCM groups of the linker. Following the conjugation of BCLH via Traut’s reagent, the slight increase in the positive ζ potential of RMSN-PEG-TLC–BCLH (RMSN-PEG-MAL + BCLH–TLC) from +10.4 to +16.6 mV, may have been due to the contribution of imine groups present in Traut’s reagent. After the conjugation of nickel to form RMSN-Ni (RMSN-PEG-TLC–BCLH + NiCl_2_), an increase in the ζ potential was noted from +16.6 to +24.2 mV due to the introduction of the positively charged nickel. Compared to MSN-PEG/PEI, the significant size increases up to 85.96 nm and the ζ potential decrease to +24.2 mV were associated with the effect and the length of the linkers used. Here, MSNs modified with longer chemical linkers were endowed with increased flexibility and reduced risk of NP surface-induced conformational changes of the conjugated proteins.

However, after conjugation of TAT-SOD, surface charges of the NPs decreased to negative charges of −12.9 (RMS-Ni-TAT-SODn: RMSN-Ni + native TAT-SOD) and −22.1 mV (RMS-Ni-TAT-SODd: RMSN-Ni + denatured TAT-SOD), meaning that the negative charge of the protein was capable of shielding the positive charge of MSN-Ni. Previous evidence suggested that Apo-SOD1 (the denatured form) had a stronger negative net charge than Holo-SOD1 (the native Cu, Zn-SOD1),^52^ which was consistent with the ζ potential variation, and also verified the successful denaturation of SOD by urea. Figure 2e also confirms structural changes of RMSN-Ni-TAT-SODd owing to the denaturation of the protein. The average weight percent of nickel functionalized onto the NPs was 0.45 wt % as determined by the ICP–MS analysis (Table S1). The protein concentration quantified by the BCA protein assay kit revealed that the weight percentages of TAT-SOD and NPs were around 6.62 wt % (Table S1). The molar ratio of nickel over the TAT-SOD protein amount was estimated to be approximately 1:3.7.

In addition to the DLS-based characterization of the NPs in suspension using Zetasizer Nano ZS, the NP tracking analysis (NTA), also based on light scattering, has emerged as a method offering direct visualization and size measurement, as well as providing valuable information regarding NP concentrations in liquid suspension. As shown in Figure 3d, the NTA method was employed to characterize NP sizes, showing the excellent dispersion of RMSN-Ni-TAT-SODn and RMSN-Ni-TAT-SODd with respective dominant sizes of 109 and 108 nm. These results were similar to the size determined with the Zetasizer Nano. From the NTA, the estimated concentrations of NPs were 1820 × 10^18^ ± 4.5 × 10^6^ and 1790 × 10^18^/mg ± 1.6 × 10^7^ NPs for RMSN-Ni-TAT-SODn and RMSN-Ni-TAT-SODd, respectively. The size and size homogeneity of the NPs are critical issues in determining the NPs’ fate, such as cellular delivery pathways. Large-sized and aggregated NPs tend to undergo internalization predominantly via phagocytosis, a cell uptake pathway by specialized immune cells and thus can be subjected to rapid clearance from the bloodstream before reaching the target site.^53^ Understanding the precise concentration of NPs is essential in controlling delivery, especially for evaluating biological differences among NPs. In this study, the NTA method demonstrated that the concentrations of RMSN-Ni-TAT-SODn and RMSN-Ni-TAT-SODd were similar and thus met the requirements for biological exploration.

### 3.2 Cellular Internalization of RMSN-Ni-TAT-SOD

**Figure 4:**
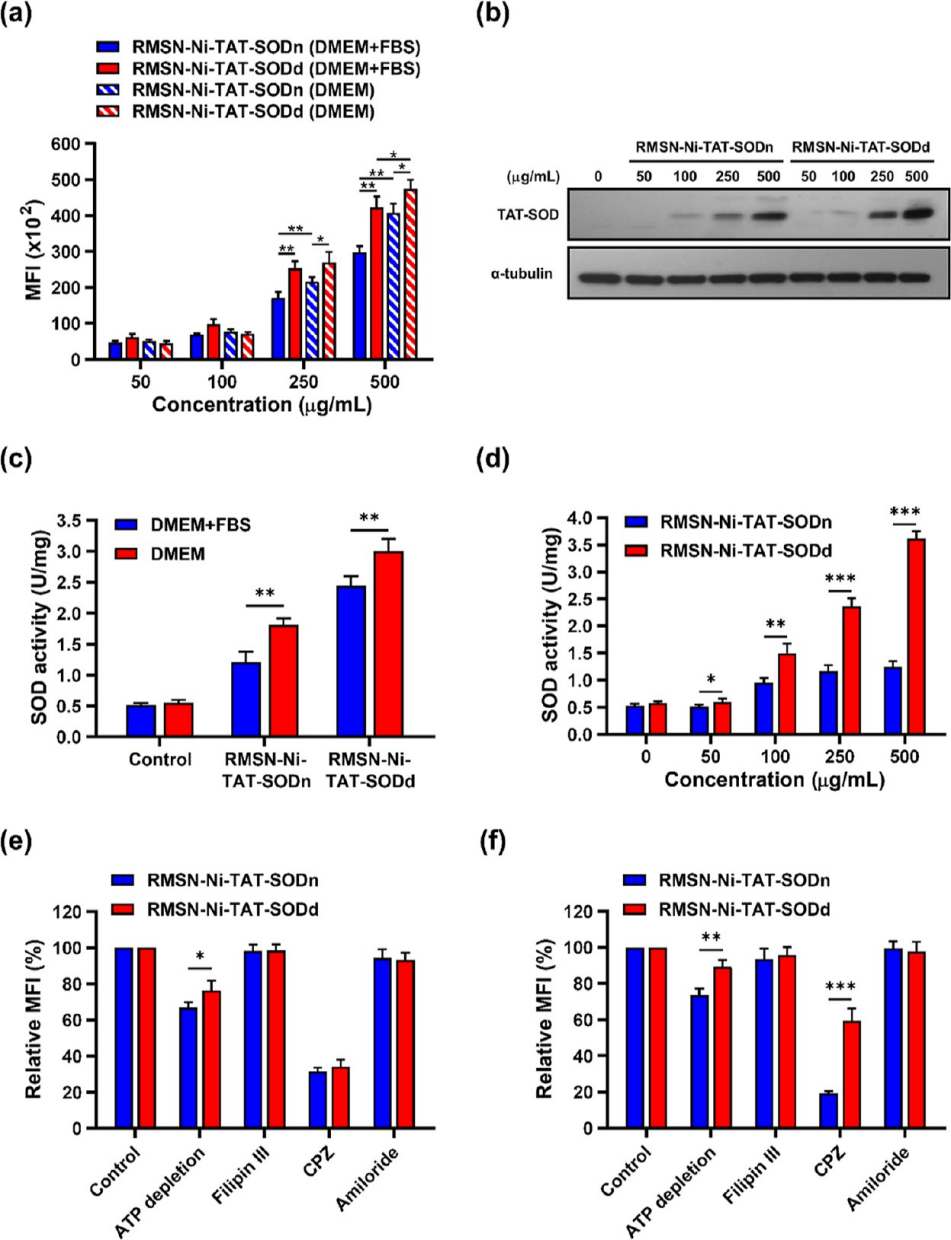
Internalization and enzymatic activity of RMSN-Ni-SOD in HeLa cells in the presence and absence of serum conditions. Various concentrations of the native (SODn) and denatured (SODd) forms of RMSN-Ni-TAT-SOD (at 50, 100, 250, and 500 μg/mL) were used to treat HeLa cells for 4 h in serum-containing and serum-free media. (a) A quantitative assessment of cell uptake was performed by a flow cytometric analysis. (b) Expression of the TAT-SOD protein by western blotting after treatment in the serum-containing medium. α-Tubulin was used as a loading control. (c) The enzymatic activity of SOD in HeLa cells treated with 250 μg/mL of RMSN-Ni-SODn or RMSN-Ni-SODd for 24 h in serum-containing and serum-free media. (d) The enzymatic activity of SOD in HeLa cells treated with different concentrations of RMSN-Ni-SODn or RMSN-Ni-SODd for 24 h in serum-containing media. (e,f) Endocytosis pathway analysis of RMSN-Ni-SODn and RMSN-Ni-SODd in HeLa cells for 4 h. Cells were preincubated with endocytosis pathway inhibitors for 1 h before incubation with 500 μg/mL of RMSN-Ni-SODn or RMSN-Ni-SODd in serum-containing (e) and serum-free (f) media. ATP depletion: sodium azide (10 mM) and 2-deoxyglucose (6 mM); caveolae-mediated endocytosis: filipin III (5 μg/mL); clathrin-mediated endocytosis: CPZ (30 μM); micropinocytosis: amiloride (10 μM). *p < 0.05; **p < 0.01; ***p < 0.001.

To evaluate the cell delivery efficiency, the red fluorescent signal of RMSNs was quantitatively detected by flow cytometry. HeLa cells were treated with different concentrations of the native and denatured forms of RMSN-Ni-TAT-SOD (at 50, 100, 250, and 500 μg/mL) in serum-containing and serum-free media for 4 h. Results presented in Figure S2 show that both forms of MSNs could be transduced into cells with almost 100% cell uptake efficacy in both culture conditions. However, quantitatively, the mean fluorescence intensity (MFI) of the intracellular delivery of the native and denatured forms of RMSN-Ni-TAT-SOD dose-dependently increased (Figure 4a). Interestingly, the denatured form, RMSN-Ni-TAT-SODd, had a higher MFI than that of the native form, RMSN-Ni-TAT-SODn, in HeLa cells at concentrations of 250 and 500 μg/mL in serum-free culture conditions. Thus, we wondered about the relationship between cell uptake and SOD levels after intracellular delivery. To evaluate the amount of SOD delivered inside cells, HeLa cells were treated with different concentrations of the native and denatured forms of RMSN-Ni-TAT-SOD (at 50, 100, 250, and 500 μg/mL) for 4 h in serum-containing media. Cells were then harvested and lysed with RIPA lysis buffer containing 500 mM of imidazole, which can break the linkage between Ni-NTA and 6× His-tag. Hence, the SOD protein could be eluted from the surface of MSNs for western blot analysis to detect cellular levels of SOD after delivery. α-Tubulin was used as a loading control. Both forms of RMSN-Ni-TAT-SOD were successfully transduced into HeLa cells in dose-dependent manners (Figure 4b). Compared to the native form, RMSN-Ni-TAT-SODn, a higher amount of SOD was detected in cells when treated with 250 and 500 μg/mL of the denatured form, RMSN-Ni-TAT-SODd, in serum-containing medium. There was no significant difference at concentrations of 50 and 100 μg/mL. In brief, the high SOD level was related to better delivery efficiency when treated with RMSN-Ni-TAT-SODd, consistent with the MFI results in Figure 4a. Next, we studied the enzymatic activity of SOD delivered by RMSN-Ni-TAT-SODd. According to our previous studies,^38,39,48^ with the intracellular delivery of the denatured form of SOD conjugated onto the MSN surface, SOD could be refolded and its biological activity restored. Chaperone proteins, including copper chaperone for SOD and heat shock protein 70, contribute to the protein folding process in the cytosol.^54^ To evaluate the serum protein effect on SOD enzymatic activity, 250 μg/mL of the native and denatured forms of RMSN-Ni-TAT-SOD were delivered into cells in serum-containing and serum-free media conditions for 24 h. Then, cell lysates were isolated, and SOD enzyme activity was assayed.

As shown in Figure 4c, RMSN-Ni-TAT-SODd displayed enzymatic activity in both serum-containing and serum-free media conditions, indicating that SODd could be successfully refolded with the help of the chaperone proteins inside cells through the unfolded protein response. Moreover, SOD delivery in serum-free media conditions revealed enzymatic activity superior to that observed with serum-containing media, implying that absorption of serum proteins onto MSNs may have significantly influenced SOD enzymatic activity. It was also observed that the delivery of RMSN-Ni-TAT-SODd presented higher enzymatic activity than RMSN-Ni-TAT-SODn in both serum protein-containing and serum-free media conditions. These results suggest that the denatured form of a protein was beneficial for protein delivery with better enzymatic activity. The enhanced SOD enzymatic activity was attributed to the increased delivery efficacy of the SODd protein, which was consistent with the cell uptake results at dosages of 250 and 500 μg/mL in Figure 4a. In addition to delivery efficacy, the conformational change/misfolding of proteins caused by the NP surface effect and protein corona absorption are critical issues influencing a protein’s biological activity when conjugated onto NPs. As shown in Figure 4d, SOD enzymatic activity increased in a dose-dependent manner when delivered in the denatured form, RMSN-Ni-SODd, in serum-containing media conditions. It was worth mentioning that the enzymatic activity of SOD delivered by RMSN-Ni-SODn was limited even at the highest concentrations of 250 and 500 μg/mL. However, RMSN-Ni-SODn showed dose-dependent cellular uptake (Figure 4a). Evidence suggests that conformational changes of proteins on the surface of NPs could change the cell uptake route^27,30^ and result in easy aggregation, leading to biological degradation by lysosomal proteases or the ubiquitin-proteasome system.^55^ This may be one of the reasons which explain the limited enzymatic activity of RMSN-Ni-SODn. Here, we propose that the denatured protein had less steric hindrance and no conformational change issues caused by the NP surface effects and may thus have avoided protein corona adsorption, leading to effective delivery and decreased degradation. With this denatured protein delivery strategy, denatured proteins can be successfully refolded, and their enzymatic activity dose-dependently restored through chaperone assembly after entering cells. Notably, SOD activity with treatment using 250 μg/mL of RMSN-Ni-SODn was around 5- (serum) to 6-times (serum-free) higher than that of control cells (Figure 4d) and superior to SOD activity in our previous reports^38,39^ with a shorter linker design, which was around just 2–3-times (serum-free) higher than control cells at the same concentration. In addition, as shown in Figure S2, it was observed that all treatment groups presented 100% cellular uptake efficacy (at 50, 100, and 250 μg/mL). However, the cellular uptake was not higher than 80% and presented a dose-dependent increase, when treated with the same concentrations in our previous report.^39^ As mentioned above, MSNs surface-modified with a longer cross-linker could promote remarkable protein activity owing to the increase in expected protein flexibility and enhanced efficacy of intracellular delivery.

Furthermore, we wondered about the cellular internalization of RMSN-Ni-SODd and RMSN-Ni-SODn and sought to understand cell uptake via different endocytosis routes. Various chemical inhibitors of endocytosis pathways, including sodium azide and 2-deoxyglucose (inhibitors of ATP depletion), filipin III (an inhibitor of caveolae-mediated endocytosis), CPZ (an inhibitor of clathrin-mediated endocytosis), and amiloride (an inhibitor of micropinocytosis), were employed to evaluate the internalization of RMSN-Ni-SODn and RMSN-Ni-SODd by HeLa cells. After pre-incubating the endocytosis inhibitors for 1 h, HeLa cells were treated with 500 μg/mL of RMSN-Ni-SODn and RMSN-Ni-SODd in serum-containing (Figure 4e) and serum-free (Figure 4f) medium conditions for another 4 h. Results showed that non-endocytosis (energy-independent) and clathrin-mediated endocytosis (energy-dependent) routes were mainly involved in the cellular uptake of RMSN-Ni-SODn and RMSN-Ni-SODd. By blocking all of the endocytosis pathways by depleting ATP, cellular uptake of RMSN-Ni-TAT-SODd and RMSN-Ni-TAT-SODd was still possible, indicating that the TAT peptide provided a non-endocytosis (energy-independent) route that contributed to cellular uptake. Also, RMSN-Ni-TAT-SODd had slightly better cellular uptake than did RMSN-Ni-TAT-SODn, especially in the serum-free medium condition, implying that the denatured form of the protein had minor steric hindrance and might alter the composition of protein corona adsorption related to the cell uptake efficiency. Only the CPZ group showed a remarkably reduced cellular uptake level to around 80% for both forms of RMSN-Ni-TAT-SOD, revealing that the internalization pathway was energy-dependent through clathrin-mediated endocytosis. However, significantly increased cellular uptake of RMSN-Ni-SODd was observed in a serum-free medium condition (∼60%) compared to a serum-containing medium condition (∼38%), demonstrating the influence of the presence of serum proteins.

These results demonstrated that serum protein adsorption caused discrepant effects on the internalization of RMSN-Ni-SODn and RMSN-Ni-SODd by cells. The energy-independent cell uptake of NPs may have partly been due to the TAT peptide and the denatured structure of SOD with less steric hindrance and high Gibbs free energy. In general, the absorption of serum proteins onto the surface of NPs can reduce cell uptake due to the effect of limited TAT exposure. Obviously, owing to the 3D native conformation of TAT-SOD limiting exposure to TAT, the TAT in RMSN-Ni-SODn was unable to interact directly with cell membranes. In contrast, RMSN-Ni-SODd, with the advantages of reduced steric hindrance, no protein conformational change issues, and avoidance of protein corona adsorption, allowed higher exposure of the positively charged TAT peptide to cell membranes, even in the presence of serum protein adsorption. In RMSN-Ni-SODd-mediated denatured TAT-SOD delivery, the TAT peptide not only facilitated the efficient delivery and non-endocytosis (energy-independent) uptake but also avoided endosomal/lysosomal degradation, which was beneficial for enhanced SOD activity. Therefore, the non-endocytosis (energy-independent) and clathrin-mediated endocytosis (energy-dependent) pathways dominated the internalization of RMSN-Ni-SODn and RMSN-Ni-SODd by HeLa cells in serum-containing and serum-free medium conditions.

### 3.3 Protein Corona Effect on RMSN-Ni-TAT-SOD Delivery

**Figure 5:**
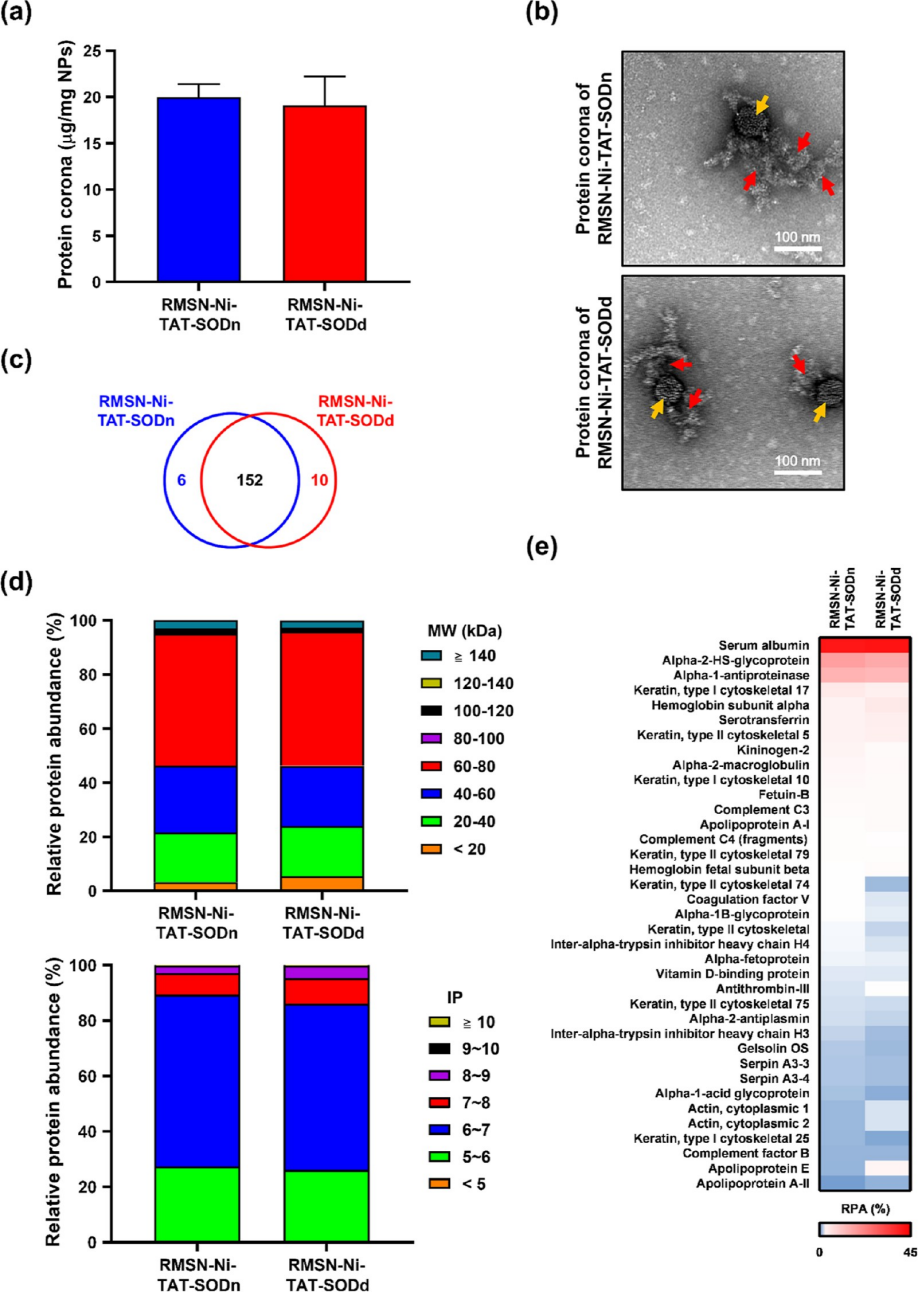
Characterization and identification of the protein corona. RMSN-Ni-TAT-SOD at 0.5 mg was incubated with DMEM containing 10% FBS for 30 min at room temperature, enabling the formation of a hard protein corona. (a) Amounts of the protein corona of RMSN-Ni-TAT-SODn and RMSN-Ni-TAT-SODd were measured by a BCA protein quantification assay. (b) TEM visualization of the protein coronas of RMSN-Ni-TAT-SODn and RMSN-Ni-TAT-SODd with negative staining by uranyl acetate. NPs, yellow arrow; protein corona, red arrow. Scale bar: 100 nm. (c) Venn diagram reporting the number of proteins in the corona adsorbed onto RMSN-Ni-TAT-SODn and RMSN-Ni-TAT-SODd according to an LC–MS analysis. (d) Classifications of corona proteins of RMSN-Ni-TAT-SODn and RMSN-Ni-TAT-SODd according to their molecular weights (MWs) and isoelectric points. (e) Heatmap illustrating the most abundant corona proteins of RMSN-Ni-TAT-SODn and RMSN-Ni-TAT-SODd.

The protein corona influences the internalization of NPs by cells and the subsequent NPs’ fate, such as cell uptake behavior, biodistribution, and cytotoxicity. Here, we studied the protein corona effect on MSN-Ni-TAT-SOD, focusing on the amount of the protein corona by a BCA analysis, the thickness of the protein corona layer by TEM observations, and the corona protein profile by the LC–MS analysis. MSN-Ni-TAT-SOD (0.5 mg) was incubated with DMEM containing 10% FBS for 30 min at room temperature, enabling a hard protein corona to form for the following experiments. Amounts of the protein corona of MSN-Ni-TAT-SODn and MSN-Ni-TAT-SODd as quantified by the BCA were estimated to be approximately 19.94 and 19.10 μg protein/mg NPs, respectively (Figure 5a). There was no significant difference between them, suggesting that the native or denatured conformation of TAT-SOD conjugated onto the MSNs did not influence the adsorption or amount of serum proteins. To visualize the absorption of the protein corona, TEM was employed.^56^Figures 5b and S3 show images of the protein corona layer surrounding the NPs after negative staining with uranyl acetate. TEM images of the protein corona presented a visual feature, such as in the case of the glycoprotein corona of SARS coronavirus via negative staining,^57^ and the corona was not in a regular or homogeneous distribution around the NPs as observed in other reports.^56,58,59^ Given the irregular layer on the NP surface, the protein corona might not wholly interfere with the biological effect of surface functionalization, such as targeting ligands and biomolecules. In our case, in some regions of the NPs without the protein corona, the TAT peptide could directly interact with the cell membrane, resulting in its effective delivery.

Proteins bound to the NPs were extracted and loaded onto 12% SDS-PAGE. The composition of corona proteins was identified using LC–MS/MS with in-gel digestion. Figure S4 shows the protein corona profile around MSN-Ni-TAT-SODn and MSN-Ni-TAT-SODd visualized by Coomassie blue staining. Basically, total numbers of 158 and 162 individual proteins were, respectively, detected in the corona of MSN-Ni-TAT-SODn and MSN-Ni-TAT-SODd. In total, 152 proteins were seen to be common to the two coronas, as shown in a Venn diagram of Figure 5c. Furthermore, the corona proteins’ relative abundances (RPAs) were calculated according to eq 1 described in “Materials and Methods”. The RPAs of identified proteins were classified based on their MW and isoelectric point (pI), and the results are presented in Figure 5d. It was noted that various MW proteins were adsorbed onto MSN-Ni-TAT-SODn and MSN-Ni-TAT-SODd to form the protein corona. Still, only a slight difference was observed, especially for proteins with MWs below 20 kDa. Also, RPAs of corona proteins based on their pI values showed a slightly noticeable difference in the range of pI 6 to pI 9. All identified corona proteins are listed in Supporting Information 2, and the top 20 most abundant corona proteins were ranked and are summarized in Table 1, which shows a slight difference in the corona fingerprints. Furthermore, a heatmap of corona proteins (only proteins with a minimum RPA of 0.1% in the corona of one of the NPs at least are shown) was created, highlighting the remarkable difference in the RPAs of corona proteins between MSN-Ni-TAT-SODn and MSN-Ni-TAT-SODd (Figure 5e).

**Table 1:** Top 20 Most Abundant Proteins in the Corona of RMSN-Ni-TAT-SODd and RMSN-Ni-TAT-SODn Identified by LC–MS/MS

top 20 abundant corona proteins of RMSN-Ni-TAT-SODn	RPA (%)	top 20 abundant corona proteins of RMSN-Ni-TAT-SODd	RPA (%)
serum albumin	41.12	serum albumin	41.59
α-2-HS-glycoprotein	17.13	α-2-HS-glycoprotein	15.20
α-1-antiproteinase	13.61	α-1-antiproteinase	12.80
keratin, type I cytoskeletal 17	4.04	hemoglobin subunit alpha	4.27
hemoglobin subunit alpha	2.59	serotransferrin	3.30
serotransferrin	2.49	keratin, type II cytoskeletal 5	3.04
keratin, type II cytoskeletal 5	2.46	keratin, type I cytoskeletal 17	2.89
kininogen-2	2.26	apolipoprotein E	2.10
α-2-macroglobulin	1.63	α-2-macroglobulin	1.28
keratin, type I cytoskeletal 10	1.45	kininogen-2	1.22
fetuin-B	1.20	keratin, type I cytoskeletal 10	1.19
complement C3	0.94	apolipoprotein A-1	1.10
apolipoprotein A-1	0.84	fetuin-B	1.08
complement C4 (fragments)	0.83	complement C3	1.06
keratin, type II cytoskeletal 79	0.78	hemoglobin fetal subunit beta	0.95
hemoglobin fetal subunit beta	0.51	keratin, type II cytoskeletal 79	0.80
keratin, type II cytoskeletal 74	0.44	complement C4 (fragments)	0.56
coagulation factor V	0.41	antithrombin-III	0.35
α-1 β-glycoprotein	0.36	α-fetoprotein	0.30
keratin, type II cytoskeletal 71	0.33	α-1 8-glycoprotein	0.29

To our knowledge, this is the first study to explore the protein corona effects on the same protein in native and denatured states when conjugated onto NPs. There is limited information that explains why both MSN-Ni-TAT-SODn and MSN-Ni-TAT-SODd can adsorb similar quantities of serum proteins with only a slight difference in protein corona fingerprints; however, a significant difference in the RPAs was observed. We herein propose that the RPA difference between them may be attributed to a difference in the ζ potentials (Figure 3c). Much fundamental research needs to be conducted in the future.

**Figure 6:**
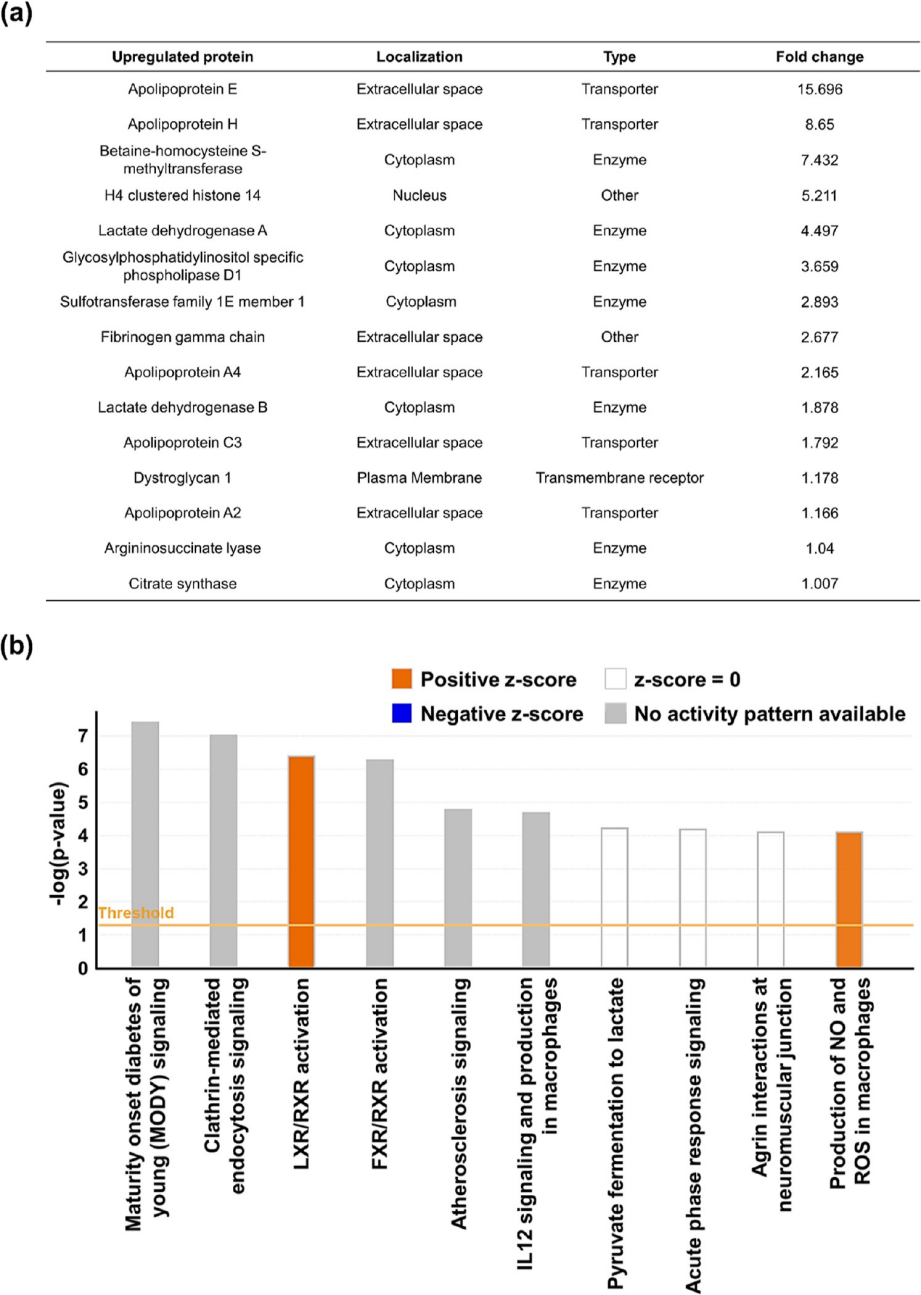
Ingenuity pathway analysis (IPA) of the protein corona of RMSN-Ni-TAT-SODd. Fold changes of corona proteins between RMSN-Ni-TAT-SODd and RMSN-Ni-TAT-SODn were analyzed by IPA software. (a) Significantly upregulated proteins in the corona of RMSN-Ni-TAT-SODd. (b) The top 10 enriched canonical pathways by the fold change of corona proteins.

To understand the molecular functions of the identified corona proteins, LC–MS/MS data were further processed according to eqs 1 and 2 and submitted to an IPA software analysis. Fold changes in the RPAs of corona proteins between RMSN-Ni-TAT-SODd and RMSN-Ni-TAT-SODn were analyzed. Significantly upregulated proteins in the corona of RMSN-Ni-TAT-SODd and their functions and localization are presented in Figure 6a. Among them are some important molecular transporters, including apolipoprotein E, β-2-glycoprotein 1 (apolipoprotein H), apolipoprotein A4, apolipoprotein C3, and apolipoprotein A2, which are associated with cell membrane crossing. Apolipoproteins are specialized in lipid transport into the cytosol via scavenger receptors expressed on cell membranes,^60^ that contribute to NP entry into cells through endocytosis.^20,31^Figure 6b depicts the top 10 enriched IPA canonical pathways according to percentage changes of the protein corona. Clathrin-mediated endocytosis signaling was the second most significant enriched mechanism involved in molecular transport into the cytosol. This result is consistent with the endocytosis pathways analysis in Figure 4e,f.

### 3.4 Biocompatibility Assessment of RMSN-Ni-TAT-SOD

**Figure 7:**
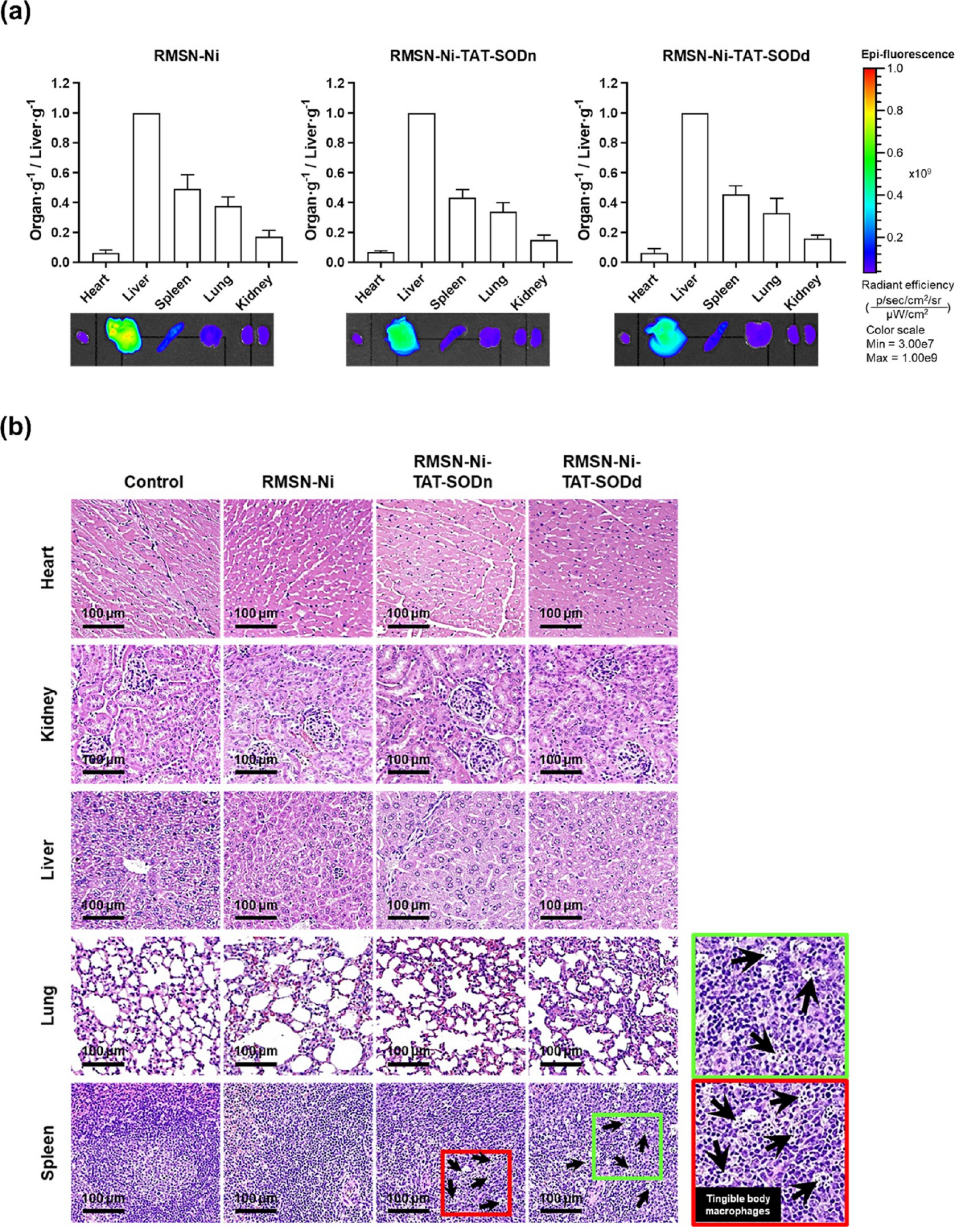
Biosafety and toxicity evaluations of RMSN-Ni-TAT-SOD. At 24 h post-injection of NPs at a dose of 50 mg/kg of body weight, mice organs were excised, and the following experiments were performed to investigate the biosafety. (a) Biodistribution of RMSN-Ni-TAT-SOD captured by the IVIS. The radiant efficiency of the RITC signal was divided by each organ’s weight and then plotted based on the ratio of each organ to the liver (organ/liver). (b) Histological analysis of different organs by H&E staining. Black arrows: lymphocyte apoptosis. Scale bar: 100 μm.

To gain further insights into the biosafety of RMSN-Ni-TAT-SOD for future clinical applications, the biodistribution, histopathological analysis, and hematotoxicity were verified in BALB/c mice. Mice (*n* = 3) were euthanized at 24 h post-IV injection of NPs (50 mg/kg). The major organs, including the heart, liver, spleen, lungs, and kidneys, were excised for the following experiments. First, the biodistribution of NPs was examined by measuring the accumulation of RITC fluorescence signals in the organs with the IVIS (Figure 7a). Not surprisingly, NPs mainly accumulated in the liver, which is the organ responsible for recognizing and clearing NPs by the mononuclear phagocyte system, such as Kupffer’s cells, leading to the enhancement of the IVIS signal. Furthermore, the amount of the NPs accumulated in each organ was estimated against the accumulation in the liver. When the total radiant efficiency (TRE) of the RITC signal per weight of each organ was divided by the TRE per liver weight, the profile of the ratio of each organ to the liver signal (organ/liver) was similar within all groups. These results demonstrated that the denatured and native forms of the SOD protein conjugated onto MSNs did not significantly influence the overall biodistribution, similar to the MSN-Ni. Interestingly, the MSN-Ni had stronger intensities in the mononuclear phagocyte system’s organs (liver and spleen) than RMSN-Ni-TAT-SODn and RSM-Ni-TAT-SODd, revealing that protein conjugation might decrease capture by the mononuclear phagocyte system. Histopathological analysis was then performed on tissue sections of different organs stained with H&E to determine whether RMSN-Ni-TAT-SOD caused tissue damage or lesions. Micrographs of all groups after treatment are presented in Figure 7b, indicating a normal histological structure in the heart, liver, lungs, and kidneys but not in the spleen. As noted with lymphocyte apoptosis (black arrows), pathological abnormalities were notably observed in the spleen treated with RMSN-Ni-TAT-SODd and RMSN-Ni-TAT-SODn compared to the control group.

At the end of the experiments, mouse peripheral blood was collected by cardiac puncture in an ethylenediaminetetraacetic acid-coated tube for a hematotoxicity study (Table 2). First, a hematological assay of the CBC was performed. After treatment with RMSN-Ni-TAT-SOD, most hematological parameters were similar to those of control mice or were still within the reference range.^61,62^ However, platelet and lymphocyte counts had obviously decreased and were also out of the reference range. Combining the results of the histopathological analysis in Figure 7b, lymphocyte apoptosis in the spleen was responsible for the abnormal parameter of lymphocytes, clearly proving an effect on the spleen’s functionality due to RMSN-Ni-TAT-SOD exposure. Further study to address the issue is of great significance. Next, the organotoxicity was investigated for liver and kidney functions by a serum biochemical assay. Levels of certain enzymes and proteins, including glutamic pyruvic transaminase, glutamic oxaloacetic transaminase, alkaline phosphatase, total bilirubin, and lactate dehydrogenase, were measured as indicators of liver function. Renal function was checked mainly through albumin, blood urea nitrogen, creatinine, and TP. Overall, none of the parameters of any treated groups significantly changed compared to control mice, and all were also in a normal reference range. No injury to the liver or kidney was noted in mice receiving MSN-Ni-TAT-SOD, proving that MSN-Ni-TAT-SOD had mainly accumulated in the liver and a small amount in the kidneys, which had not affected organ functionality. Overall, the impact of RMSN-Ni-TAT-SOD demonstrates the potential of the denatured protein delivery approach for in vivo protein therapy.

**Table 2:** Hematological and Blood Biochemical Parameters of BALB/c Mice Treated with RMSN Derivatives upon IV Injection^a^

test		control	RMSN-Ni (150 mg/kg)	RMSN-NI-TAT-SODn (150 mg/kg)	RMSN-Ni-TAT-SODd (150 mg/kg)
CBC	TP (g/dL)	4.97 ± 0.09	5.07 ± 0.05	3.33 ± 2.36	5.2 ± 0.14
	ALB (g/dL)	2.23 ± 0.05	2.33 ± 0.05	2.3 ± 0.16	2.33 ± 0.05
	WBC (10^9^/L)	4.663 ± 0.442	6.353 ± 0.298	4.327 ± 0.36	4.554 ± 0.32
	NEU (10^9^/L)	0.62 ± 0.13	0.897 ± 0.172	0.933 ± 0.381	0.62 ± 0.041
	LYM (10^9^/L)	3.863 ± 0.539	5.217 ± 0.122	1.21 ± 0.079**^,^^b^	1.607 ± 0.182**^,^^b^
	MONO (10^9^/L)	0.07 ± 0.016	0.113 ± 0.019	0.04 ± 0.008	0.087 ± 0.009
	EOS (10^9^/L)	0.107 ± 0.012	0.127 ± 0.021	0.027 ± 0.005	0.037 ± 0.005
	BASO (10^9^/L)	0.003 ± 0.005	0 ± 0	0.007 ± 0.005	0.003 ± 0.005
	RBC (10^9^/L)	10.493 ± 0.284	10.6 ± 0.149	10.403 ± 0.3	11.057 ± 0.037
	HGB (g/dL)	16.2 ± 0.36	16.5 ± 0.22	16.13 ± 0.58	17.17 ± 0.05
	MCV (fL)	49.03 ± 0.29	49.4 ± 0.29	49.07 ± 0.34	49.5 ± 0.08
	MCH (pg)	15.4 ± 0.08	15.57 ± 0.05	15.5 ± 0.14	15.53 ± 0.05
	PLT (10^9^/L)	646.7 ± 29	514.3 ± 122.4	285.3 ± 33***^,^^b^	229.3 ± 5.9***^,^^b^
biochemistry	GOT (U/L)	68 ± 1.7	46 ± 3.74	71.7 ± 9.1	90.7 ± 5
	GPT (U/L)	78 ± 7.3	52 ± 8.5	48.7 ± 3.7	68.3 ± 8
	ALP (U/L)	222 ± 10.6	197 ± 5.1	105.7 ± 10.5	103.7 ± 7.3
	TBIL (mg/dL)	<0.1	<0.1	<0.1	<0.1
	LDH (U/L)	1189 ± 125.5	1202 ± 117	1207.7 ± 169.3	1449 ± 165.1
	ALB (g/dL)	2.23 ± 0.05	2.33 ± 0.05	2.3 ± 0.16	2.33 ± 0.05
	BUN (mg/dL)	30.3 ± 1.7	24.3 ± 0.9	23 ± 1.8	21.7 ± 0.9
	CRE (mg/dL)	0.23 ± 0.05	0.17 ± 0.05	0.2 ± 0	0.2 ± 0
	TP (g/dL)	4.97 ± 0.09	5.07 ± 0.05	3.33 ± 2.38	52 ± 0.14

a Values are the mean ± SD. ** *p* < 0.01 and *** *p* < 0.001 compared to control mice.

b Lower than the reference range.

### 3.5 Potential Application of RMSN-Ni-TAT-SOD in Neuron Therapy

Currently, a therapeutic approach for neuron diseases, such as neurodegenerative diseases, has been developed that mainly focuses on gene delivery to enhance neuron differentiation and small-molecular drugs to protect neuron cells from oxidative stress. Nevertheless, the efficiency of gene transfection and the efficacy of drugs targeting neuron cells present significant issues that limit their therapeutic application. Direct protein delivery into neuron cells is one of the promising options, thus providing a potential opportunity to overcome the drawbacks of current therapeutics for neurodegenerative diseases. The therapeutic approach presented herein that combines MSNs and a denatured protein delivery system is eagerly anticipated.

**Figure 8:**
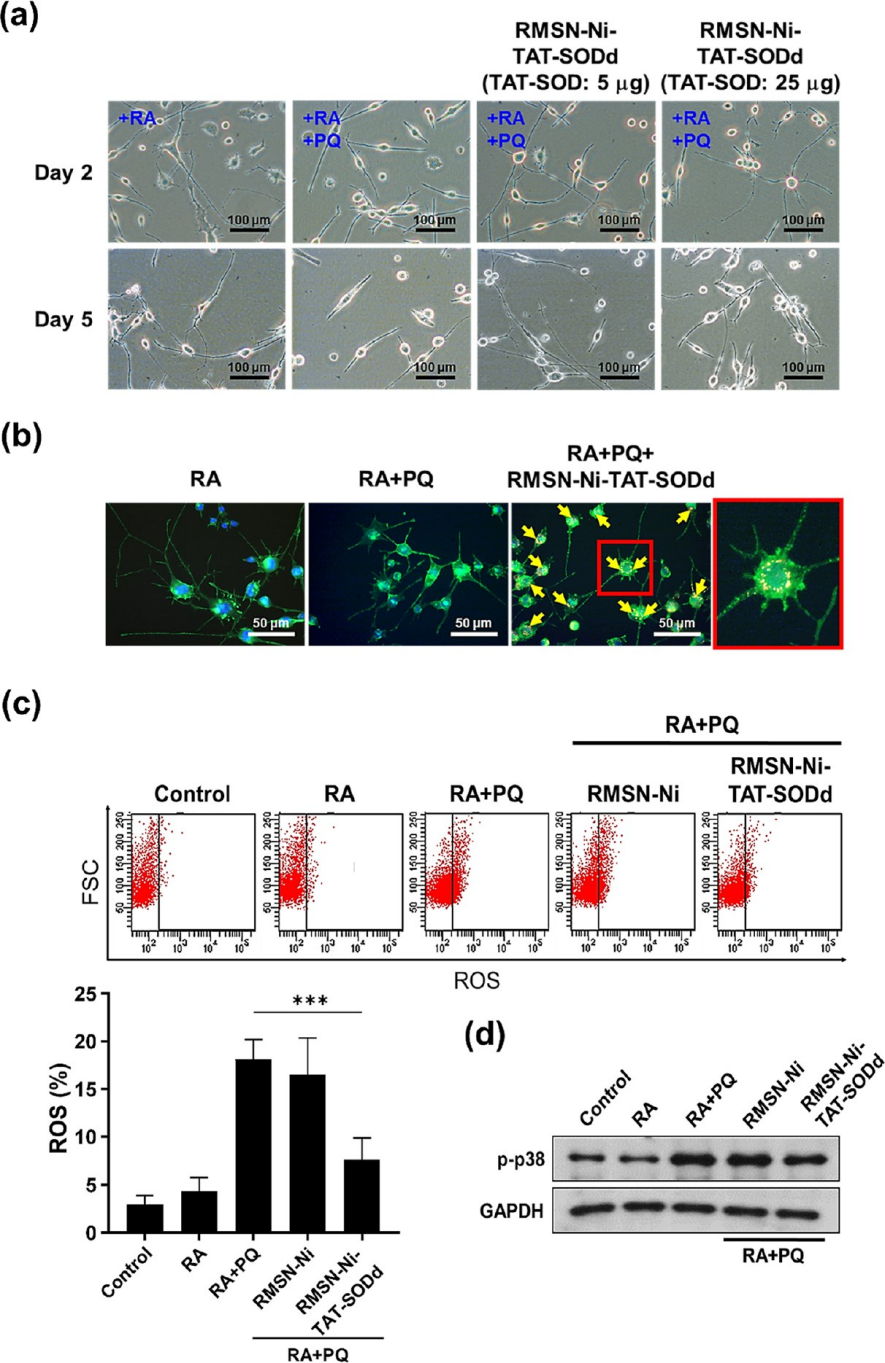
RMSN-Ni-TAT-SODd enhanced neurite outgrowth against ROS attack in N2a cells. N2a cells were treated with the denatured form, MSN-Ni-TAT-SODd, (equivalent to 5 and 25 μg of TAT-SOD) for 4 h and then stimulated with and without RA (20 μM) and PQ (30 μM). (a) The bright-field images of N2a cells after 2 and 5 days of treatments with RA, PQ, and RSN-Ni-TAT-SODd. Scale bar: 100 μm. (b) Fluorescent microscopic images of N2a cells treated with RA, PQ, and RMSN-Ni-TAT-SODd on day 8. Blue: DAPI-stained nuclei; green: Alexa Fluor 488-phalloidin-stained cytoskeleton. Yellow arrow: RMSN-Ni-TAT-SODd localization (RMSN-Ni-TAT-SODd overlapping with the cytoskeleton). Under the same conditions, N2a cells were treated with RMSN-Ni (equivalent to the weight of MSN-Ni-TAT-SODd) and RMSN-Ni-TAT-SODd (equivalent to 25 μg of TAT-SOD). Scale bar: 50 μm. (c) Intracellular ROS stained by DHE (5 μM) and quantified by a flow cytometric analysis. ***p < 0.001. (d) Western blotting detection of the protein expression level of phosphorylated (p)-p38.

RA is a well-known derivative of vitamin A and acts as a specific modulator with a promoting effect on neuronal differentiation and neurite growth.^63^ PQ is a widely used herbicide that can induce ROS that cause cellular toxicity.^64^ The delivery of therapeutic proteins with a neuroprotective effect to neuron cells is a potential therapeutic approach to neuron diseases. Hence, we further evaluated the possibility of using the MSN-mediated denatured protein delivery approach, which could protect neurite outgrowth under a PQ-induced ROS condition. First, N2a cells were treated with the denatured form, MSN-Ni-TAT-SODd, for 4 h and then stimulated with and without RA (20 μM) and PQ (30 μM). Neurite outgrowth and neurite formation were captured by microscopy. As shown in Figure 8a, bright-field images on days 2 and 5 indicated that RA effectively promoted neuron differentiation of N2a cells, whereas PQ significantly reduced RA-induced neurite outgrowth. ROS generated by PQ obviously induced damage to N2a cells, resulting in shorter neurites on day 5. However, PQ-reduced neurite outgrowth could be reversed by treatment with MSN-Ni-SODd. Through scavenging ROS under PQ conditions, treatment with low (equivalent to 5 μg of TAT-SOD) and high (equivalent to 25 μg of TAT-SOD) concentrations of MSN-Ni-SODd fostered longer neurite lengths than PQ treatment. Neuron protection by antioxidant SOD delivery was further investigated. Figure 8b shows fluorescence microscopic imaging performed on different groups after 8 days of treatment. The cytoskeleton stained with Alexa Fluor 488-labeled phalloidin (green color) clearly distinguished the neuron morphology. Additionally, DAPI staining was used to determine nuclei (blue color). The yellow color was the overlapping of RMSN-Ni-TAT-SODd and cytoskeleton, which was responsible for the localization of RMSN-Ni-TAT-SODd (yellow arrows). There were lots of RMSN-Ni-TAT-SODd in the cytoplasm (red enlarged), clearly demonstrating that the enhanced neurite outgrowth was attributed to the effective intracellular delivery of SODd by MSNs, which protected neuron cell differentiation from PQ-induced ROS damage.

To confirm the contribution of RMSN-Ni-TAT-SODd, intracellular ROS were detected with fluorescent DHE (5 μM) and analyzed using flow cytometry. Quantitatively, the ROS presented in Figure 8c showed that PQ exposure effectively induced oxidative stress in N2a cells stimulated with RA and co-treated with RMSN-Ni (equivalent to the weight of MSN-Ni-TAT-SODd) without SOD. In contrast, the delivery of MSN-Ni-TAT-SODd (equivalent to 25 μg of TAT-SOD) significantly reduced the proportion of ROS in N2a cells. Western blotting was then employed to detect the protein level of phosphorylated (p)-p38, a biomarker of intracellular ROS-induced inflammation, confirming that higher p-p38 expression was related to PQ exposure in N2a cells (Figure 8d). A decreased expression level of p-p38 was observed after treatment with MSN-Ni-TAT-SODd (equivalent to 25 μg of TAT-SOD) plus PQ, which was consistent with the flow cytometric analysis (Figure 8c). It was noted that the MSNs were capable of intracellular delivery of SODd, and refolding was performed to enhance the enzymatic activity, followed by the elimination of ROS, enabling neurite outgrowth. We validated the MSN-mediated delivery of SODd that can serve as an attractive therapeutic approach for neuron disease therapy, highlighting its potential to improve therapeutic outcomes that are superior to the current low efficiency and limited gene and small-molecule drug therapies.

## 4 Conclusions

**Figure 9:**
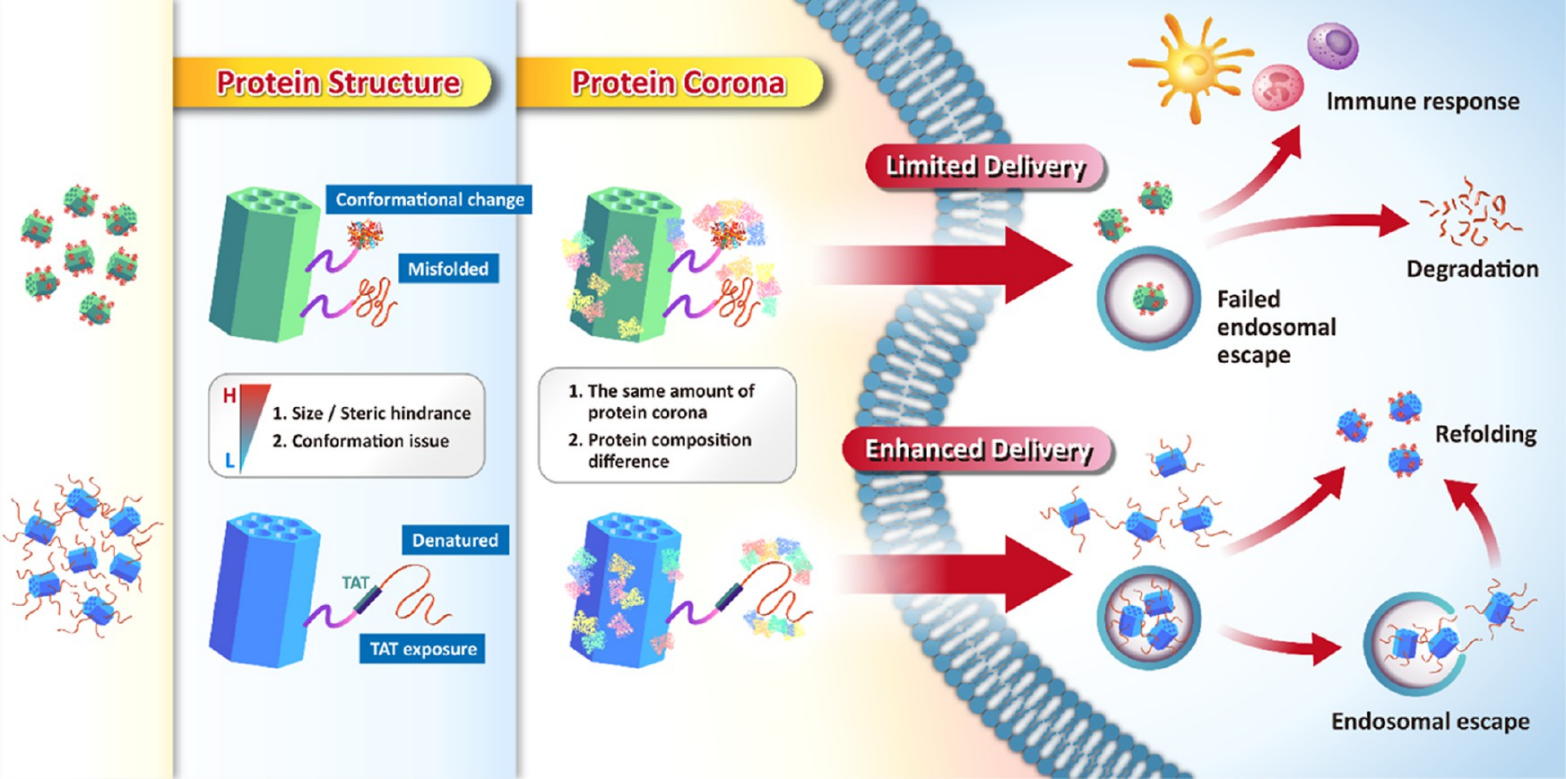
Schematic illustration of a comparison of native and denatured protein-conjugated MSNs for intracellular delivery.

In summary, we synthesized biocompatible RMSN-Ni-TAT-SOD with surface modification by grafting PEI molecules and Ni-NTA chelated ligands for native and denatured TAT-SOD protein conjugation via metal coordination. By introducing a PEG crosslinker, this functionalization created a distance with the NPs that increased the protein’s flexibility and decreased the NPs’ surface-induced secondary conformational changes. After transduction into cells, RMSN-Ni-TAT-SODd could be refolded, restoring its specific enzymatic activity thanks to protein chaperone assembly. On the contrary, SODn activity was low at high concentrations, probably due to aggregation or misfolding, leading to subsequent degradation via lysosomal proteases or the ubiquitin-proteasome system. The delivery of denatured proteins takes advantage of a reduced size/steric hindrance, no protein conformational change issues, avoidance of protein corona adsorption, and enabling exposure of the TAT peptide, which thus benefitted enhanced delivery (Figure 9). RMSN-Ni-TAT-SODd exhibited acceptable biocompatibility, demonstrating the potential for its in vivo use. On the way to overcome current challenges of the limited efficiency of gene therapy and low therapeutic efficacy of small-molecule drugs in neuron therapy, RMSN-Ni-TAT-SODd may be an attractive therapeutic approach against ROS damage to protect neuron cell outgrowth. This approach displays the opportunity to bridge the gap between scientific research and industrial production.

## Supporting Information Available

Characterization of various RMSN derivatives, stability analysis of RMSN-PEG/PEI, ζ potential, cellular uptake, TEM visualization of the protein corona layer, and SDS-PAGE of protein corona profile (PDF)
List of the total identified corona proteins (XLSX)
