# Supplementary material for: Overcoming Cytosolic Delivery Barriers of Proteins Using Denatured Protein-Conjugated Mesoporous Silica Nanoparticles
Source: ACS Appl Mater Interfaces. 2022 Dec 23;15(1):432–51. doi: 10.1021/acsami.2c17544 (PMC9896485; doi:10.1021/acsami.2c17544)
Supplement: Supplementary file 1 — am2c17544_si_001.pdf [file am2c17544_si_001.pdf]

# Supporting Information

## Overcoming Cytosolic Delivery Barriers of Proteins Using Denatured Protein-Conjugated Mesoporous Silica Nanoparticles

Julien Dembélé[1,2†], Jou-Hsuan Liao[3†], Tsang-Pai Liu[4], and Yi-Ping Chen[5,6*]

[1]Graduate Institute of Biomedical Materials & Tissue Engineering, College of Biomedical Engineering, Taipei Medical University, Taipei 11031, Taiwan

[2]Laboratory of Toxicology, Environment and Health, Doctorate School of Health, University Joseph Ki-Zerbo, 03 BP 7021 Ouaga, Burkina Faso.

[3]Department of Chemistry, National Taiwan University, Taipei 10617, Taiwan

[4]Department of Surgery, Mackay Memorial Hospital, Taipei 10449, Taiwan

[5]Graduate Institute of Nanomedicine and Medical Engineering and [6]International PhD Program in Biomedical Engineering, College of Biomedical Engineering, Taipei Medical University, Taipei 11031, Taiwan

[†] Julien Dembélé and Jou-Hsuan Liao contributed equally to this work.

* Correspondence: haychen@tmu.edu.tw

**Table S1.** Characterization of various RMSN derivatives

| Sample | DLS size (nm) | PDI[a] | Zeta potential (mV) | Ni (wt%)[b] | TAT-SOD (wt%)[c] |
|---|---|---|---|---|---|
| RMSN-PEG/PEI (PBS) | 61.56 ± 0.54 | 0.087 ± 0.011 | +40.7 ± 4.2 | – | – |
| RMSN-Ni (PBS) | 85.96 ± 0.61 | 0.169 ± 0.005 | +24.2 ± 1.2 | 0.45 | – |
| RMSN-Ni-TAT-SODn (PBS) | 113.37 ± 0.15 | 0.244 ± 0.010 | −12.9 ± 2.1 | 0.45 | 6.62 ± 0.144 |
| RMSN-Ni-TAT-SODd (PBS) | 122.46 ± 2.10 | 0.289 ± 0.020 | −22.1 ± 0.6 | 0.45 | 6.62 ± 0.144 |
| RMSN-Ni-TAT-SODn (DMEM+FBS) | 107.67 ± 4.90 | 0.331 ± 0.043 | −12.9 ± 2.1 | 0.45 | 6.62 ± 0.144 |
| RMSN-Ni-TAT-SODd (DMEM+FBS) | 113.10 ± 3.84 | 0.489 ± 0.009 | −22.1 ± 0.6 | 0.45 | 6.62 ± 0.144 |

a. Polydispersity index
b. Measured by ICP-MS analysis
c. Measured by BCA protein assay kit

**Table S2.** Stability analysis of RMSN-PEG/PEI

| Time | Size (nm) | PDI | Zeta potential (mV) |
|---|---|---|---|
| 1 week | 61.23 ± 0.30 | 0.113 ± 0.006 | +35.1 ± 0.9 |
| 3 weeks | 62.78 ± 0.31 | 0.109 ± 0.014 | +38.2 ± 0.6 |

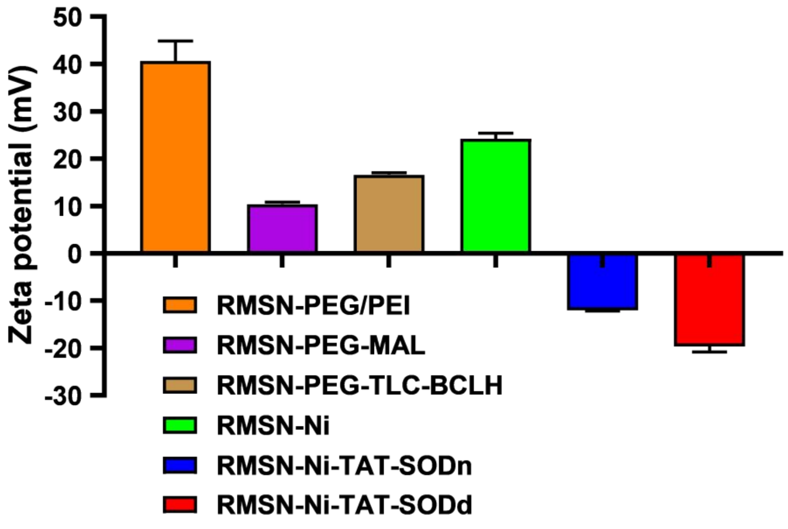


**Figure S1.** Zeta potential of various RMSN derivatives with different surface modifications.

RMSN-PEG/PEI: RMSN+PEG+PEI

RMSN-PEG-MAL: RMSN-PEG/PEI+MAL-PEG-SCM

RMSN-PEG-TLC-BCLH: RMSN-PEG-MAL+BCLH-TLC

RMSN-Ni: RMSN-PEG-TLC-BCLH+$NiCl_2$

RMS-Ni-TAT-SODn: RMSN-Ni+native TAT-SOD

RMS-Ni-TAT-SODd: RMSN-Ni+denatured TAT-SOD

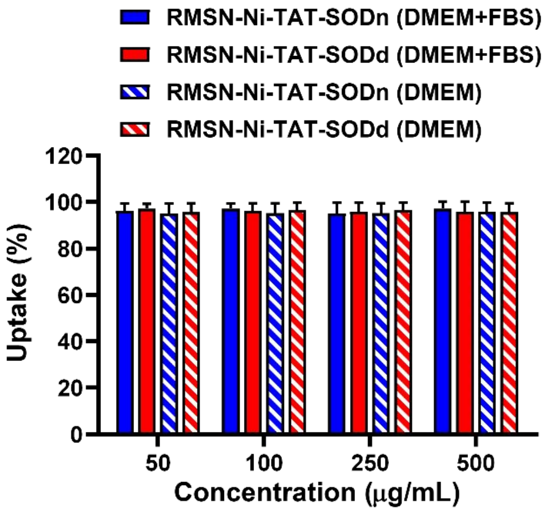


**Figure S2.** Cellular uptake of HeLa cells treated with various concentrations of RMSN-Ni-TAT-SODn and RMSN-Ni-TAT-SODd (at 50, 100, 250, and 500 μg/mL) in serum-containing and serum-free media for 4 h.

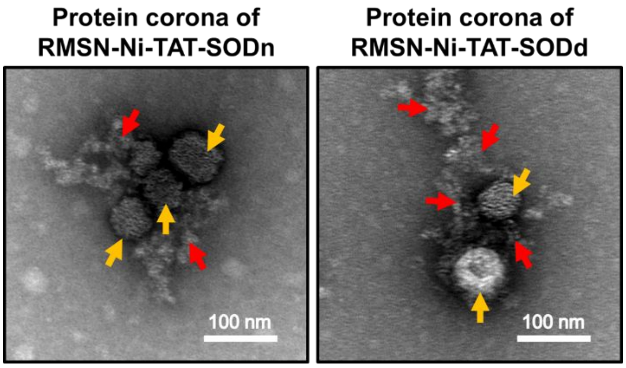


**Figure S3.** TEM visualization of the protein corona layer. (a) RMSN-Ni-TAT-SODn and (b) RMSN-Ni-TAT-SODd with negative staining by uranyl acetate. NPs, yellow arrow; protein corona, red arrow.

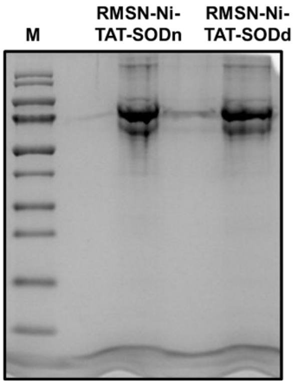


**Figure S4.** SDS-PAGE of protein corona profile on mesoporous silica nanoparticle RMSN-Ni-TAT-SODn and MSN-Ni-TAT-SODd.
